# WRKY transcription factor genes in wild rice *Oryza nivara*

**DOI:** 10.1093/dnares/dsw025

**Published:** 2016-06-26

**Authors:** Hengjian Xu, Kenneth A. Watanabe, Liyuan Zhang, Qingxi J. Shen

**Affiliations:** ^1^School of Life Sciences, Shandong University of Technology, Zibo 255000, Shandong Province, People’s Republic of China; ^2^School of Life Sciences, University of Nevada Las Vegas, Las Vegas, NV 89154, USA

**Keywords:** WRKY transcription factors, Genome, Wild rice, *Oryza nivara*

## Abstract

The *WRKY* transcription factor family is one of the largest gene families involved in plant development and stress response. Although many *WRKY* genes have been studied in cultivated rice (*Oryza sativa*), the *WRKY* genes in the wild rice species *Oryza nivara*, the direct progenitor of *O. sativa*, have not been studied. *O. nivara* shows abundant genetic diversity and elite drought and disease resistance features. Herein, a total of 97 *O. nivara WRKY* (*OnWRKY*) genes were identified. RNA-sequencing demonstrates that *OnWRKY* genes were generally expressed at higher levels in the roots of 30-day-old plants. Bioinformatic analyses suggest that most of *OnWRKY* genes could be induced by salicylic acid, abscisic acid, and drought. Abundant potential MAPK phosphorylation sites in OnWRKYs suggest that activities of most OnWRKYs can be regulated by phosphorylation. Phylogenetic analyses of OnWRKYs support a novel hypothesis that ancient group IIc OnWRKYs were the original ancestors of only some group IIc and group III WRKYs. The analyses also offer strong support that group IIc OnWRKYs containing the HVE sequence in their zinc finger motifs were derived from group Ia WRKYs. This study provides a solid foundation for the study of the evolution and functions of *WRKY* genes in *O. nivara*.

## 1. Introduction

Since the first *WRKY* gene was reported for sweet potato in 1994,^1^ many *WRKY* genes have been identified in a wide variety of organisms.^2–9^ A large majority of *WRKY* genes function as a positive or negative regulators in plant responses to various biotic and abiotic stresses.^10^ Because stress responses of plants are often involved in hormone signalling, WRKY proteins have been demonstrated to mediate the crosstalk of hormones such as abscisic acid (ABA), gibberellins, salicylic acid (SA), jasmonic acid, and ethylene.^9^^,^^11–17^ In addition, *WRKY* genes also play a role in the control of leaf senescence and seed development.^18–23^ In these physiological processes, *WRKY* genes are regulated by multiple factors,^24^ and most of which involve phosphorylation.^25–27^ Several WRKY proteins have been shown to be phosphorylated by MAPKs. These transcription factors usually contain conserved motifs for phosphorylation by MAPKs.^28–30^

WRKY transcription factors contain one or two WRKY domains that are composed of a WRKY motif and a zinc finger motif. The core sequence of a WRKY motif is WRKYGQK with some variants including WRKYGKK, WRKYGEK, WRKYGRK, WKKYGQK, WKRYGQK, and WSKYEQK.^7^^,^^31^ Two types of zinc fingers, the C_2_H_2_ motif (C–X_4–5_–C–X_22–23_–H–X_1_–H) and the C_2_HC motif (C–X_5__–__7_–C–X_23_–H–X_1_–C), are found in WRKY proteins.^8^^,^^32^

*WRKY* genes are usually divided into three groups, groups I, II, and III.^8^^,^^32^ Group I has two WRKY domains; group II has one WRKY domain with a C_2_H_2_ zinc finger; and group III has one WRKY domain with a C_2_HC zinc finger. Group I was subsequently divided into two subgroups, Ia containing C_2_H_2_ zinc fingers and Ib containing C_2_HC zinc fingers. Group II was subsequently divided into five subgroups, IIa, IIb, IIc, IId, and IIe, based on their phylogenetic relationship.^8^^,^^32^ Current publications support the fact that WRKY group II should be divided into three subgroups, IIa + b, IIc, and IId + e, because the IIa and IIb subgroups, as well as the IId and IIe subgroups always cluster into a single clade.^2^^,^^33^ Genes with an incomplete WRKY domain were assigned to group IV.^32^

The *Oryza* genus contains 23 species and 9 recognized genome types.^34^ The AA type genome is present in *Oryza sativa* rice and its relatives, as well as the *Oryza glaberrima* and its relatives. They are thought to be an example of parallel evolution in crop plants.^34^^,^^35^ The *O.* *sativa*, as well as its close wild relatives—perennial *Oryza rufipogon* and annual *Oryza nivara*, originated in the south Asia area.^36^ *O.* *nivara*, the direct progenitor of the *O.* *sativa* L., has abundant genetic diversity^35^^,^^37^^,^^38^ and elite features of drought and disease resistance.^39–41^ Considering the diverse functions of *WRKY* genes in various physiological and developmental processes, comprehensive studies of the *WRKY* gene family in *O.* *nivara* will shed light on the evolution of this wild rice species and on the functions of this large family of transcription factors.

## 2. Materials and methods

### 2.1. Genomic datasets

The genomic data of *O.* *nivara* Sharma *et* Shastry and *O.* *sativa* L. ssp. *indica* were downloaded from GRAMENE (http://www.gramene.org/). A hidden Markov model (HMM) was constructed using the *O.* *sativa* WRKY proteins^32^^,^^42^ and then was used to identify the WRKY proteins in the *O.* *nivara* genome using HMMER 3.1b2^43^ with a cut-off *E*-value of 0.001. The orthologues were identified by comparing the WRKY sequences in *O.* *nivara* and those in *O.* *sativa* ssp. *japonica* (*Osj*) via batch BLASTp.^44^ The gene IDs of the *OnWRKYs* were determined based on their closest relatives to *O.* *sativa*.

Nomenclature of On*WRKY* genes were based on their homology to *OsjWRKY* genes. *OsWRKY* genes have had conflicting names in the past and a solution was proposed by the Committee on Gene Symbolization, Nomenclature and Linkage (CGSNL).^45^^,^^46^ We performed BLASTP queries using all 127 putative OnWRKY proteins against the 135 putative OsjWRKY proteins, and assigned names to the *OnWRKY* genes based on their closest homologous OsjWRKY proteins. This should facilitate future research of the *WRKY* genes in rice. We recommend that the same nomenclature be utilized in all *Oryzoideae* plants.

### 2.2. Classification of the *OnWRKY* genes

The full-length protein sequences of the OnWRKY proteins were aligned by the MUSCLE method embedded in MEGA6.0^47^ and classified into groups based on their WRKY domains, as in previous publications.^8^^,^^32^^,^^42^^,^^48^ In brief, OnWRKYs are divided into groups I, II, III, and IV. Group I consists of two subgroups. Subgroup Ia OnWRKYs contain two WRKY domains with C_2_H_2_ zinc fingers, and subgroup Ib OnWRKYs contains two WRKY domains with C_2_HC zinc fingers. The group II WRKY proteins contain one WRKY domain with a C_2_H_2_ zinc finger. Group II was further divided into five subgroups based on presence of specific sequences in their zinc finger motifs. Subgroup IIa contains CX_5_CPVKKK(L/V)Q motif, IIb CX_5_CPVRKQVQ, IIc CX_4_C, IId CX_5_CPARKHVE, and IIe CX_5_CPARK(Q/M)V(E/D). Group III WRKY proteins contain one WRKY domain with a C_2_HC zinc finger.^8^^,^^32^ The WRKY proteins with incomplete WRKY domains were classified as group IV.^32^

### 2.3. Phylogenetic analyses

The WRKY domains and the full-length protein sequences of the *OnWRKY* genes were used to analyse their phylogenetic relationship. The amino acid sequences were aligned using MUSCLE in MEGA 6.0 with default parameters and then adjusted manually to the best possible alignment.^47^ The unrooted neighbour-joining (NJ) phylogenetic tree was constructed based on the aligned WRKY domains (including both N-terminal and C-terminal domains) and full predicted protein sequences of the *OnWRKY* genes using MEGA 6.0 with bootstrap replications of 1000.

### 2.4. Visualization of chromosomal location and transcript structures of *OnWRKY* genes

To determine the chromosomal locations of the *OnWRKY* genes, the locus coordinates and gene sizes were extracted from genome data using an in-house PERL program. The distribution of *OnWRKY* genes on the chromosomes was analysed using MAPchart 2.3.^49^

The transcript structures and positions of the WRKY and zinc finger motifs were generated using the Transcript Structure and Domain Display software.^50^

### 2.5. Intron, exon, and motif analyses

The exons, introns, and their relative peptide sequences were extracted from the genomic data using an in-house PERL program. The positions of the intron/exon boundaries were analysed and visualized by another in-house PERL program. The intron phases in the WRKY domains were analyzed according to Xie et al.^32^ To identify other domains in the OnWRKYs, MOTIF Search program (http://www.genome.jp/tools/motif/) was used to search against the Pfam database with an *E*-value of <0.01. Other potential motifs of OnWRKYs, like MAPK phosphorylation sites, were identified based on previous publications.^28–30^

### 2.6. Genomic and RNA sequencing data of *O.* *nivara*

RNA sequencing data for the shoots and roots of the 30-day-old plants, respectively, panicles at the booting stage, and flag leaves at the booting stage of *O.* *nivara* were downloaded from SRA (accession number SRR1722159-62).^51^ The *O.* *nivara* genome sequence was downloaded from the EnsemblPlants website.^52^ Short reads were aligned and expression analysis was performed using the Tophat and Cufflinks software.^53^^,^^54^

## 3. Results and discussion

### 3.1. Identification of *OnWRKY* genes

HMM analyses led to identification of 127 putative *OnWRKY* genes in *O.* *nivara*. Among them seven (01G13710.1, 03G01380.1, 03G36380.2, 04G06040.1, 05G21470.1, 11G15870.1, and 11G15890.1) do not have a WRKY domain-coding sequence, and hence were excluded from further analyses. 03G29220.1 and 03G30350.1 contain a WKKY and WRMC motif, respectively, and were counted as OnWRKYs. For a gene with several alternative transcripts, the transcript with the longest protein sequence was used for that locus, leading to elimination of 23 alternative transcripts. Overall, 97 genes were identified, named *OnWRKY1* to *OnWRKY125* corresponding to the names of their orthologues in *O.* *sativa*. The details of these *OnWRKYs*, such as their locus numbers, types of the encoded WRKY domains and sizes of the deduced peptides, are listed in Supplementary Table S1. The lengths of the OnWRKYs range from 149 to 1,389 amino acids. The MSU and RAP gene IDs of the *O.* *sativa* ssp. *japonica* orthologs can also be found in the table. 

### 3.2. Expression analysis of *OnWRKY* genes based on the RNA-seq data

**Figure 1:**
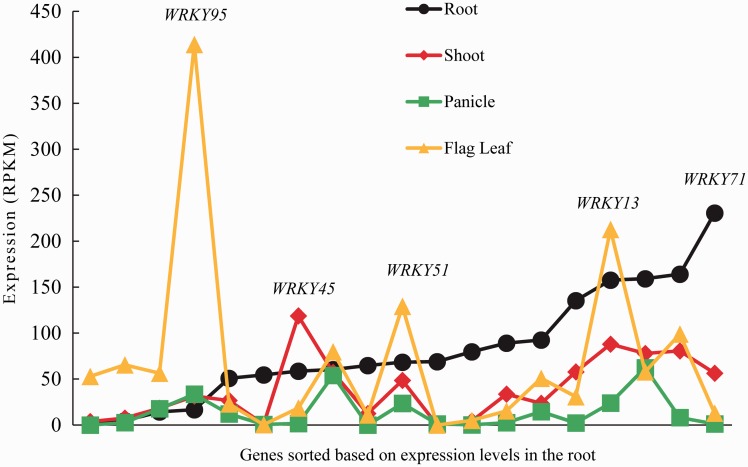
The highest expression of *OnWRKY* genes was generally detected in the root. Only genes that had an expression level of at least 50 RPKM in at least one tissue were plotted. The root and shoot samples came from 30-day-old plants and the panicles and flag leaves were from plants at the booting stage. The *OnWRKY* genes along the X-axis were sorted in ascending order of expression in the root tissue.

Publically available RNA-seq data were derived from the shoots and roots of 30-day-old plants, respectively, and flag leaves and panicles at the booting stage, respectively. Overall, the *OnWRKY* expression levels were lowest in the panicle, followed by the flag leaf, shoot, and root (Supplementary Table S2). Most *OnWRKY* genes showed some level of expression in at least one tissue. The panicle data were used to determine a baseline of expression because it had the lowest average expression level of all the plant tissues. Since >95% of the *OnWRKY* genes had an expression level of <50 reads per kilobase per million reads (RPKM) in the panicle, 50 RPKM was used as the baseline for expression. Genes with at least 50 RPKM in at least one tissue were depicted in Fig. 1. The *OnWRKY* genes were sorted from low expression to high expression based on the root data because the average *OnWRKY* gene expression level in root was the highest. As can be seen in Fig 1, there are many genes that have peak expression levels in different tissues. The gene with the highest expression was *OnWRKY95*, with an expression level of 414 RPKM in the flag leaf; its expression levels in other tissues were <40 RPKM. The gene with the next highest peak expression was *OnWRKY71*, which had an expression level of 231 RPKM in the root tissue. All other tissues had an *OnWRKY71* expression level <60 RPKM. *OnWRKY45* had the highest level of expression (119 RPKM) in the shoot, and <60 RPKM in all other tissues. *OnWRKY13* had a high expression level (>80 RPKM) in three of the plant tissues excluding the panicle.

### 3.3. Classification of the *OnWRKY* genes

**Figure 2:**
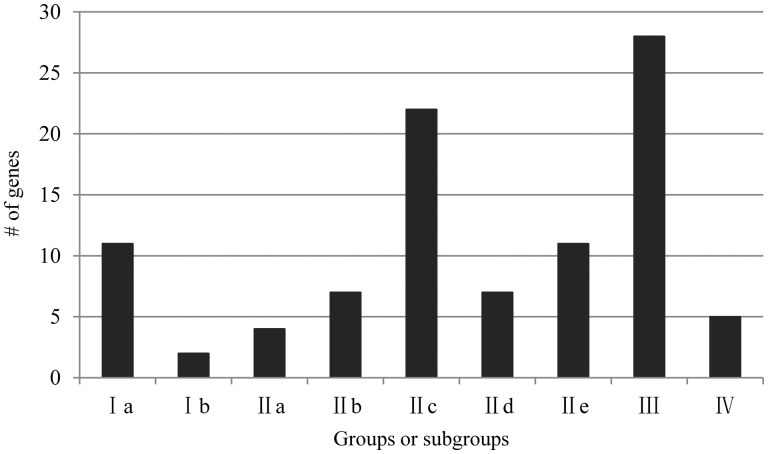
Group III is the largest group of *OnWRKY* genes. The distribution of the WRKY genes based on group or subgroup.

The *OnWRKY* genes were classified into different groups and subgroups according to published methods.^8^^,^^32^ Subgroups Ia and IIe each contain 11 genes, and IIb and IId each contain 7 genes (Fig. 2). Subgroup Ib is the smallest group, with only two genes. The largest OnWRKY group is Group III, containing 28 genes. Subgroup IIc contains 22 genes. Five OnWRKY proteins contain incomplete WRKY domains and thus belong to group IV, of which four (OnWRKY22, 26, 27, and 119) could be classified into the other subgroups (subgroups IIb, IIc, and group III) based on their zinc finger structure, and one, OnWRKY56, does not have a complete zinc finger structure and is not able to be classified into any other group.

### 3.4. Chromosomal distribution of *OnWRKY* genes

**Figure 3:**
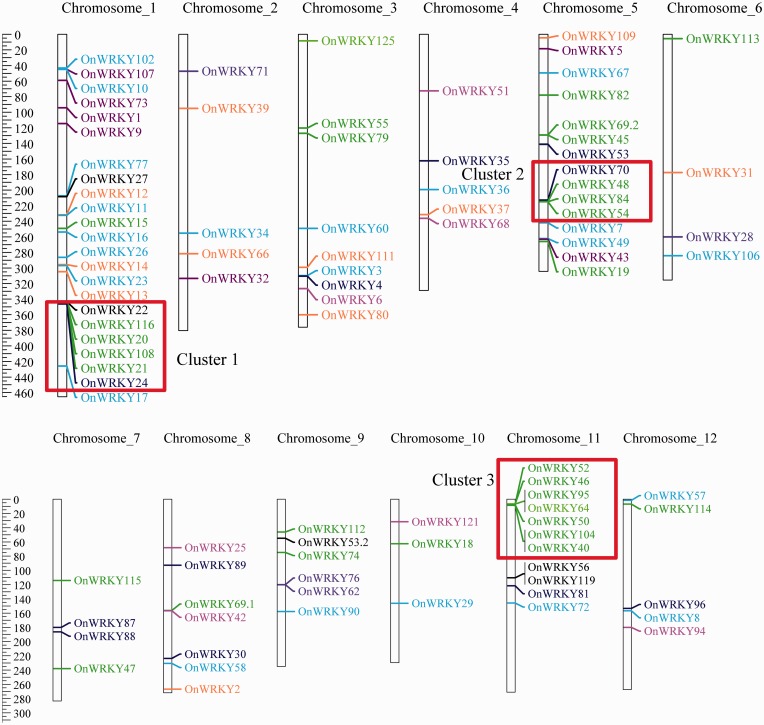
Distribution of *OnWRKY* genes on the *O. nivara* chromosomes is uneven. The vertical columns represent chromosomes with the gene names shown on the right. The unit on the ruler is 100 kbp. The chromosome sizes were reduced by a factor of 10^5^. The colour codes for genes are: blue, subgroups Ia; light green, Ib; light purple, IIa; purple, IIb; light blue, IIc; pink, IId; orange, IIe and green, III.

**Figure 4:**
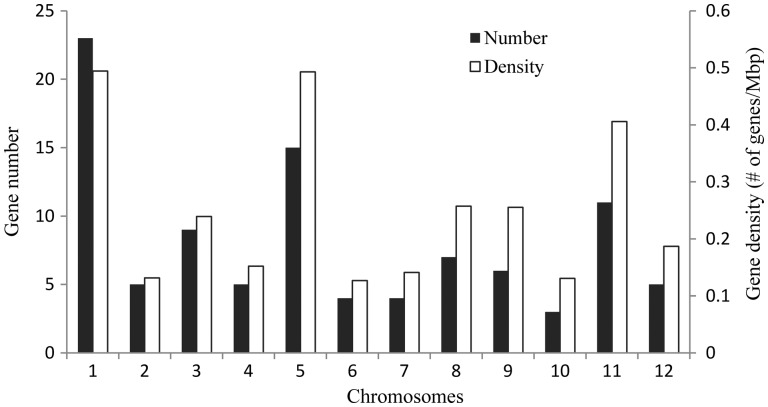
Chromosome 1 contains the largest number of *OnWRKY* genes, but shares the highest gene density with chromosome 5. The gene density was defined as gene number per million base pairs.

The *O.* *nivara* genome is an AA genome with 12 chromosomes. The number of *OnWRKY* genes on each chromosome varies (Fig. 3). Chromosome 1 has the most number of *OnWRKY* genes (23 genes), followed by chromosomes 5 and 11 (15 and 11 genes, respectively) (Fig. 4). Chromosome 10 has only three *OnWRKY* genes. The distribution of the *OnWRKY* genes on each chromosome is uneven. Some *OnWRKY* genes are clustered on chromosomes 1, 5, and 11, and some are scattered on chromosomes 2 and 6. The clustered sequences may have resulted from gene duplication events. The distribution density of *OnWRKY* genes on the chromosomes was then measured by gene number per million base pairs. As expected, the densities of *OnWRKY* genes on chromosomes 1, 5, and 11 are the highest (Fig. 4).

The distribution of *OnWRKY* genes on the chromosomes varies from group to group (Supplementary Table S3). Subgroup IIc was the most widespread group, found on 11 chromosomes. Group III and subgroup Ia were found on 10 and 8 chromosomes, respectively. They are followed by subgroup IIe and IId, located on seven and five chromosomes, respectively. Subgroups IIa and IIb were each found on three chromosomes.

### 3.5. Comparison of *OnWRKY* gene clusters to *OsWRKY* gene clusters

From visual inspection of the locations of the *OnWRKY* genes, it can be seen that there are regions where the density of *OnWRKY* genes is quite high. These regions will be referred to as “clusters” of *WRKY* genes. There are three clusters that stand out, cluster 1 on chromosome 1 (Chr1:34,313,705–34,621,563), cluster 2 on chromosome 5 (Chr5:21,272,296–21,492,695), and cluster 3 on chromosome 11 (Chr11:653,019–852,319) (Fig. 3). All three of these regions are <400,000 bp in length and contain at least four *OnWRKY* genes.

To determine whether these WRKY gene clusters are conserved, the position of *WRKY* genes on *O.* *nivara* were compared with those on *O.* *sativa* (Supplementary Fig. S1). Cluster 1 on *O.* *nivara* contained a similar corresponding region on chromosome 1 (Chr1:34,981,468–35,347,976) of *O.* *sativa* with the same six homologous *WRKY* genes (*WRKY22, 116, 20, 108, 21*, and *24*) in the same order. Four of these WRKY genes belong to group III.

Cluster 2 on *O.* *nivara* also contained a similar corresponding region on chromosome 5 (Chr5:23,310,474–23,550,612) of *O.* *sativa* with the same four homologous *WRKY* genes (*WRKY70, 48, 84*, and *54*) in the same order. Three of these WRKY genes belong to group III.

Cluster 3 on *O.* *nivara* contained a similar corresponding region on chromosome 11 (Chr11:749,998–793,116) of *O.* *sativa*, however, there were fewer *OsWRKY* genes. *O.* *nivara* contained seven *WRKY* genes (*WRKY52, 46, 95, 64, 50, 104*, and *40*), six of which belong to group III, while *O.* *sativa* contained only five *WRKY* genes (*WRKY52, 46, 104, 40*, and *50*). The order of the *WRKY* genes was also not consistent when comparing the last three *OnWRKY* genes to the *OsWRKY* genes. *OsWRKY95* and *64* were missing from the cluster and were located in a different cluster on chromosome 12.

*Oryza sativa* contains a cluster of *WRKY* genes that does not exist in *O.* *nivara*. The region Chr12:789,403–836,516 (cluster 4) on *O.* *sativa* contains five *OsWRKY* genes (*WRKY114, 97, 95, 64*, and *65*). All of these are group III WRKY genes. *O.* *nivara* only has one *WRKY* gene in the corresponding location and that is *OnWRKY114*. *O.* *nivara* does not contain *OnWRKY97* or *OnWRKY65*.

Most of the WRKY genes within the above-mentioned clusters are group III, the largest group of *WRKY* genes. This supports previous suggestions that group III *WRKY* genes expand more quickly than other *WRKY* genes.^7^ The close similarity of the first three clusters validates *O.* *nivara* as a progenitor of *O.* *sativa*. The *O.* *sativa* cluster 4 developed later during the evolution of *O.* *sativa*. One possibility is that a gene relocation event occurred by which *OsWRKY95* and *64* relocated from chromosome 11 to chromosome 12. These genes are very closely spaced with about 1,300 bp between them making this a plausible possibility. After the relocation event, these genes replicated to form the other nearby group III WRKY genes *OsWRKY97* and *65* resulting in a new cluster. This is validated by the fact that *O.* *nivara* does not have a corresponding *OnWRKY97* or *OnWRKY65*. However, the fact the *O.* *nivara* contains *OnWRKY114* on chromosome 12 in a similar location suggests that *OsWRKY114* was already present on chromosome 12. Maybe the relocation of *OsWRKY95* and *64* just happened to land near *OsWRKY114*. Further research of this phenomenon is necessary to determine the exact sequence of events.

### 3.6. Structures of *OnWRKY* genes

**Figure 5:**
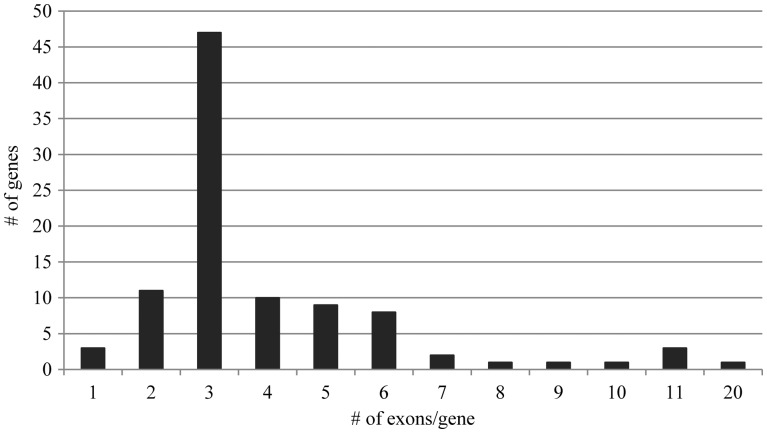
Most of *OnWRKY* genes contain two to six exons. The distribution of exon number of the WRKY genes ranges from 1 to 20 with three exon genes being the most abundant.

The number of exons and introns in the *OnWRKY* genes varies from 1 to 20 (Fig. 5), with 47 *OnWRKY* genes (48%) containing 3 exons. About 88% of *OnWRKY* genes contain two to six exons. Three *OnWRKY* genes contain 1 exon and one contains 20 exons. Interestingly, although three exon genes are most common, all of group I *OnWRKY* genes contain more than three exons.

**Figure 6:**
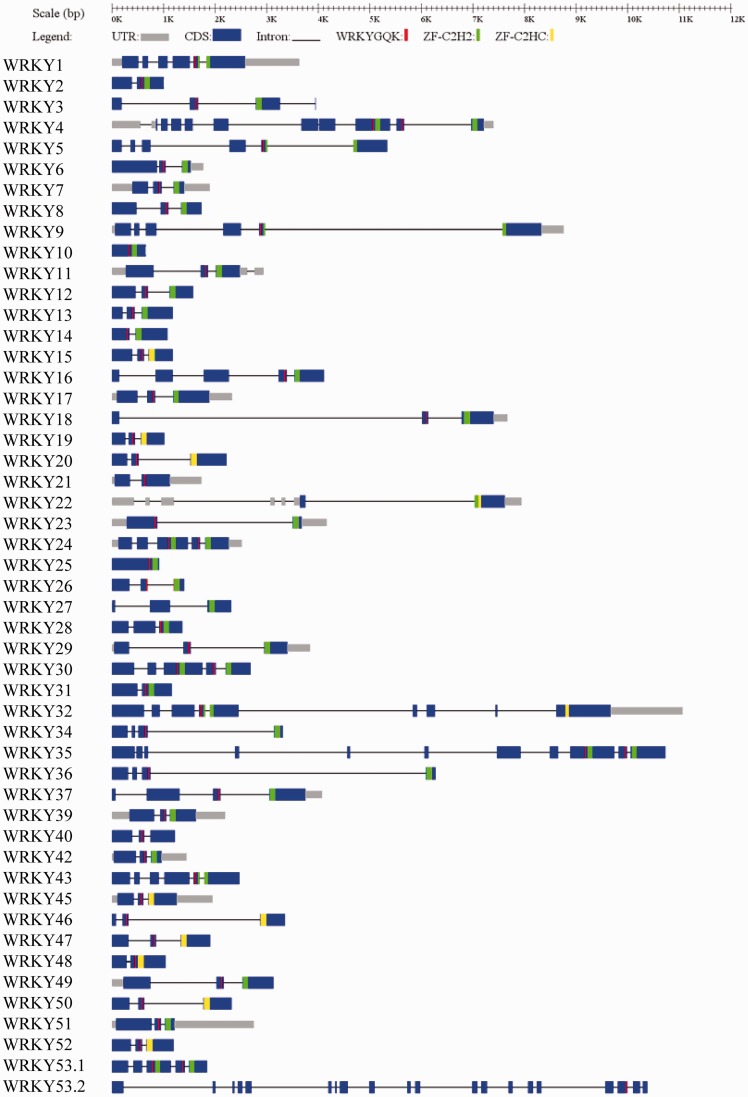

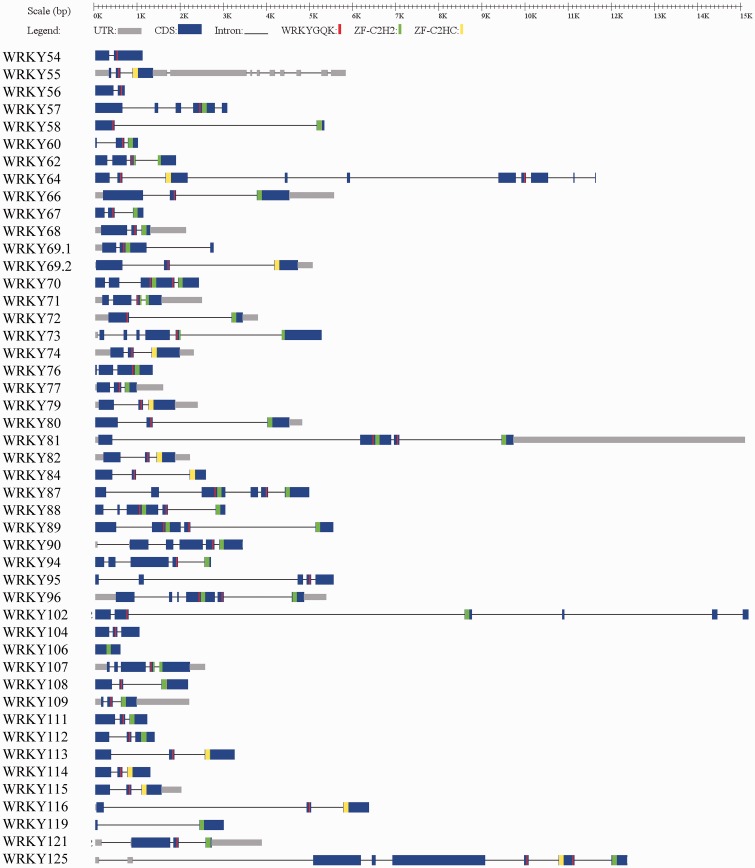
The exon-intron structures of *OnWRKY* genes. The exons and introns are indicated by blue rectangles and black lines, respectively. The untranslated regions (UTRs) are indicated by grey rectangles. The WRKY motifs are represented by red vertical bars. The C2H2 zinc fingers are represented by green bars. The C2HC zing fingers are represented by yellow bars.

The OnWRKY transcripts structures were generated for visualization of the exons, introns, and positions of the WRKY and zing finger motifs (Fig. 6).^50^ Analyses of introns within the sequences encoding the WRKY domains support the group classification. The DNA sequences encoding C-terminal WRKY domains of the subgroup Ia *OnWRKY* genes contain PR type introns, which are inserted in the codon of an arginine residue in the WRKY domain (Supplementary Fig. S2),^8^^,^^32^^,^^55^ but no N-terminal WRKY domains contain PR type introns. This suggests that the current N-terminal WRKY domains might not be direct duplications of the C-terminal WRKY domains. On the contrary, regions encoding both C-terminal and N-terminal WRKY domains of subgroup Ib genes have PR type introns. The PR type intron also occurs in the sequences encoding WRKY domains of subgroups IIc, IId, IIe, and III *OnWRKY* genes.

Our previous work assigned introns inserted between two codons as phase 0, those between the first and second nucleotides of a codon as phase 1, and those between the second and third nucleotides of a codon as phase 2. All PR type introns are phase 2 except *OnWRKY58*, which is phase 1. The VQR type introns (introns located directly before the codon of the valine residue in the WRKY domain) were observed in the sequences encoding WRKY domains in subgroups IIa and IIb genes. The VQR type introns all are phase 0. Sixteen WRKY domains harbour neither of the two types of introns. This reflects the complex evolution of *OnWRKY* genes.

### 3.7. Motif and domain analyses of OnWRKY proteins

Most OnWRKY proteins have a conserved “WRKY” motif, but some had a variant motif such as WKKY, WVKY, and WRMC. The sequence W(R/K)(K/R)Y was recommended as the consensus sequence for the WRKY motif.^32^ The question remains whether the WRMC or WVKY motifs can function as WRKY motifs. For example, the OnWRKY proteins (OnWRKY106 and −125) have variant WRKY motifs WRMC and WVKY, respectively, but also contain the conserved zinc finger motifs. Similarly, the heptamer WRKYGQK is present in the WRKY domain of 82% of OnWRKY proteins. Other variants such as WRKYGKK (OnWRKY7, −10, −26, −67, and −77), WKKYGQK (OnWRKY60), and WRMCGQK (OnWRKY106) were found in subgroup IIc OnWRKY proteins. WRKYGEK is found in six group III OnWRKY proteins (OnWRKY18, −46, −52, −55, −84, and −114). WRKYGLK and WVKYGQK were exclusively found in the subgroup Ib OnWRKY125 protein. The motif WRKYGKK was reported having lost the ability of binding to the W-box in soybean.^56^

*WRKY* genes mediate plant responses to various abiotic and biotic stresses.^57^ Determination of conserved domains on *OnWRKYs* could help elucidate their functions. Extra domains were found in OnWRKY proteins by using the MOTIF Search program (Supplementary Table S4). Subgroup Ib OnWRKY125, the longest OnWRKY protein with 1,389 amino acids, contains 2 WRKY domains and all motifs listed in Supplementary Table S4 except FAR1, FLYWCH, Plant_zn_clust and Mito_fiss_reg. Interestingly, OnWRKY125 also contains an NB-ARC domain (with an *E*-value of 2.4 × 10^−^^5^). The genes that contain both NB-ARC and WRKY domain coding sequences were classified as *RWRKY* genes by Rinerson et al.^55^ Two *RWRKY* genes (*OsjRWRKY1* and *2*) were found in *O. sativa* subs *japonica* and one (*OsiRWRKY1*) in *O. sativa* subs *indica*.^55^ Protein BLAST queries of these three OsWRKYs against the deduced peptide sequences of *O. nivara* identified *OnWRKY125* and *ONIVA11g15890* as potential *RWRKY* genes. Upon examination of the protein sequence of *ONIVA11g15890*, no WRKY domain was identified and though it does contain an NB-ARC domain, it cannot be classified as a RWRKY gene. NB-ARC domains have also been identified in *Arabidopsis thaliana*, *Fragaria vesca*, *Glycine max*, *Sorghum bicolor*, *Setaria italic*, and *Theobroma cacao*.^55^ The NB-ARC functions as a signalling domain for plant resistance.^58–60^ Presence of the AAA+ domains suggest that the function of OnWRKY125 might be ATP dependent.^61^ The Thymidylate_kin domain in OnWRKY125 implies its role in regulating the cell cycle.^62^

FLYWCH zinc finger domains were found in subgroup IId OnWRKY6 and group III OnWRKY22, −82, and −95. The FLYWCH domain was first discovered in Drosophila and plays a putative role in protein–protein interactions.^63^ Another domain Plant–zn–clust^59^ only occurs in subgroup IId OnWRKYs with *E*-values ranging from 10^−^^11^ to 10^−^^20^, suggesting the special function of subgroup IId OnWRKYs. FAR1 domains were found in subgroup IIc OnWRKY7 and −67. The FAR1 domain is involved in the phyA-signalling pathway.^64^^,^^65^ It suggests that OnWRKY67 function in the phytochrome signalling pathway. Mito_fiss_reg domain is presence in subgroup IIa OnWRKY71, which is related to mitochondrial fission.^66^^,^^67^ *OsWRKY71* was reported to function in signalling pathway in aleurone cells,^11^^,^^32^ and the OsWRKY subgroup IIa transcription factors also have been shown to modulate rice innate immunity.^68^ Mito_fiss_reg domain may help to explore its functional mechanism. This information could help elucidate the function and evolutionary relationships of these *OnWRKY* genes.

MAPK phosphorylation is involved in rice resistance to pathogens.^69^ Several WRKYs have been reported to be phosphorylated by MAPKs in tobacco, *Arabidopsis* and rice.^70–74^ MAPK phosphorylation sites usually contain some signature motifs.^28–30^ Potential MAPK phosphorylation sites in OnWRKY proteins were analysed. About 90% of OnWRKYs contain at least one putative MAPK phosphorylation sites (Supplementary Fig. S3). The maximum number of putative phosphorylation sites in one protein is 9 (in OnWRKY35). Ten OnWRKY proteins contain no phosphorylation sites, of which seven OnWRKY proteins belong to subgroup IIc. These results demonstrate that phosphorylation might be a popular regulation process for *OnWRKY* genes.

The substrate motif that interacts with MAPK is called the docking motif (D-motif, D-site, and D-domain).^30^^,^^75^^,^^76^ For MAPKs, substrate specificity is ensured through the use of the D-motif. About 65.5% of the OnWRKY proteins with phosphorylation sites contain one to nine putative D-motifs. Another type of docking motif was reported as the F-site,^76^^,^^77^ which was not found in OnWRKYs. Forty OnWRKYs do not contain any type of docking motifs, among which six OnWRKYs do not contain phosphorylation sites. These OnWRKYs might contain other types of docking motifs. Abundant MAPK phosphorylation sites found in OnWRKYs indicate that most OnWRKYs could be regulated by MAPK.

### 3.8. Phylogenetic analysis of the OnWRKY domains

**Figure 7:**
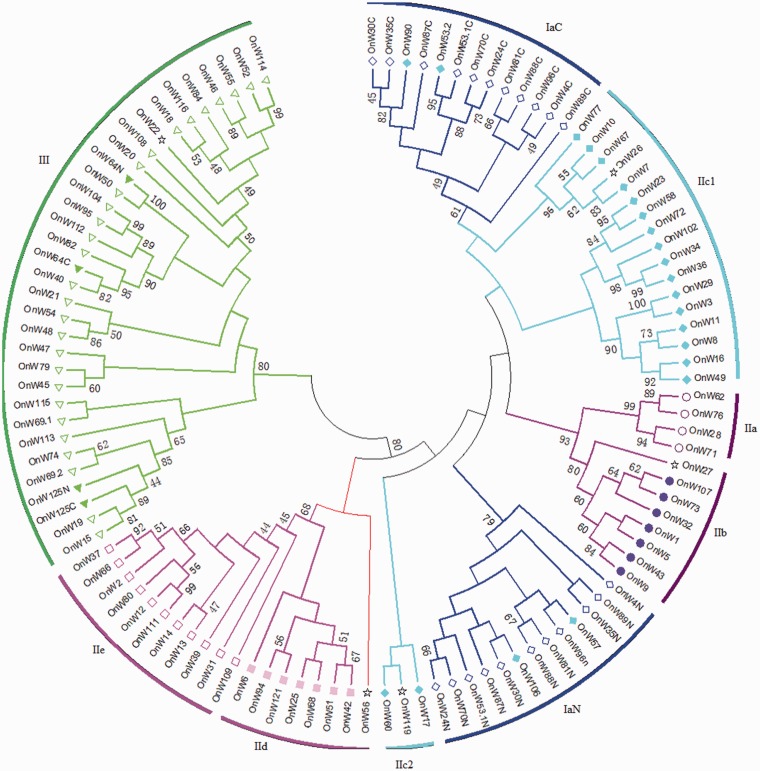
Phylogenetic analyses of the WRKY domains of OnWRKYs. The sequences were aligned by MUSCLE in the MEGA6 using the default parameters. The consensus NJ_tree was shown with the results of 1,000 bootstrap replications. Bootstrap values (≥60) are displayed in nodes. Group Ia: hollow diamond; Group Ib: filled triangle; Group IIa: circle; Group IIb: disc; Group IIc: filled diamond; group IId: filled square; Group IIe: square; Group III: triangle; Group IV: star.

The phylogenetic relationship between the OnWRKY domains was examined by using MEGA6.0 for the multiple sequence alignment of all OnWRKY domains with bootstrap analyses. Similar to what was found in other plants,^8^^,^^32^ OnWRKYs clustered into different clades of the NJ tree (Fig. 7). The N-terminal domains of subgroup Ia OnWRKYs clustered into clade IaN and C-terminal domains of subgroup Ia were clustered into another clade (IaC). Group II diverged into four clades, of which subgroups IIa and IIb clustered into one clade, subgroups IId and IIe clustered into another, and subgroup IIc clustered into two clades, IIc1 and IIc2 (Fig. 7). Our results supported the previous proposal to merge the subgroups IIa and IIb into a single subgroup, as well as subgroups IId and IIe into a single subgroup.^32^ Most of the subgroup IIc *OnWRKY* genes clustered into the IIc1 clade that neighbours the IaC clade. The IIc1 clade was not strongly supported by bootstrapping. Two group IIc OnWRKY domains (OnWRKY53.2 and −90) clustered within the IaC clade and another two (OnWRKY57 and −106) within the IaN clade, which demonstrates a close evolutionary relationship between the subgroup IIc and subgroup Ia genes. The domains of three subgroup IIc OnWRKYs (17, −60, and −119) clustered into the IIc2 clade. This suggests a paralytic evolution of subgroup IIc domains. The WRKY domains of Group III OnWRKYs were clustered into one clade that includes the WRKY domains of all subgroup Ib OnWRKYs, which is strongly supported by a high bootstrap value (80). Group IV OnWRKYs were scattered amongst the different clades. The group IV OnWRKY56 fell just outside of clade IId + e, suggesting it possibly belongs to this subgroup. In brief, all clades are supported by high bootstrap values (≥60), except for subgroup IIc.

### 3.9. Phylogenetic analysis of full-length OnWRKY proteins

**Figure 8:**
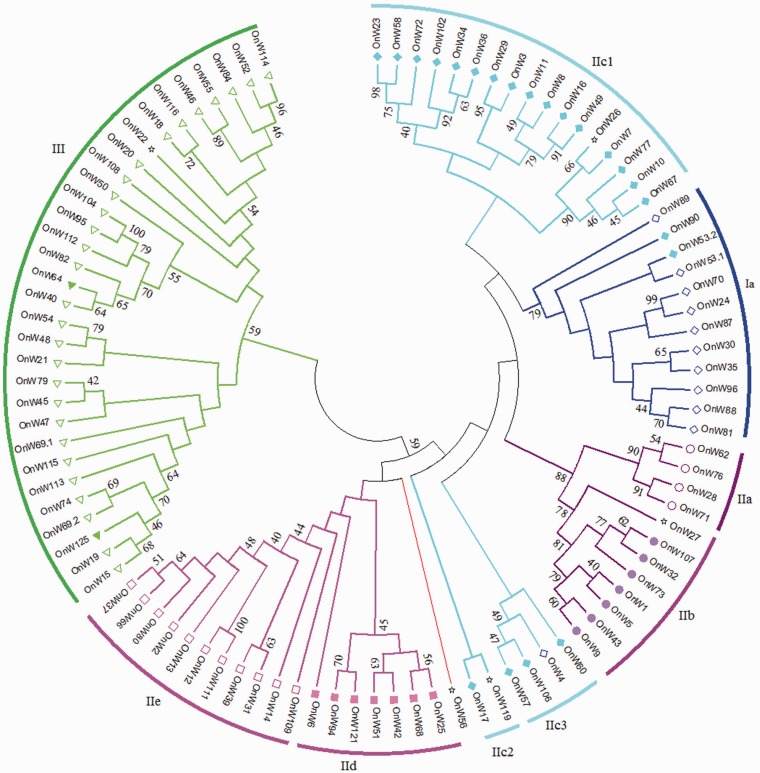
Phylogenetic analyses on full-length OnWRKY proteins. The sequences were aligned by using the MUSCLE method in MEGA6 using the default parameters. The consensus NJ_tree was shown with the results of 1,000 bootstrap replications. Bootstrap values (≥60) are displayed in nodes. Group Ia, diamond; Group Ib, filled triangle; Group IIa, circle; Group IIb, filled circle; Group IIc, filled diamond; Group IId, filled square; group IIe, square; group III, Triangle; Group IV: star.

The phylogenetic tree made from just the WRKY domains may miss important information on the evolution of *OnWRKY* genes. Hence, an NJ phylogenetic tree was generated using the full-length OnWRKY proteins. As shown in Fig. 8, the full-length phylogenetic tree is similar to the domain tree (Fig. 7). The subgroup Ia is clustered into one clade except for OnWRKY4, and subgroups IIa + b, IId + e, and III + Ib each clustered into one clade. Subgroup IIc diverged into three clades, IIc1, IIc2, and IIc3. The largest is the IIc1 clade, which neighbours clade Ia, but the other two clades of subgroup IIc (IIc2 and IIc3) were located in between clades IIa + b and IId + e. However, only clades Ia and IIa + b are supported by higher bootstrap values (≥60). This neighbour joining tree is further supported by a maximum likelihood tree (Supplementary Fig. S4).

The differences between these two trees are mainly on subgroups Ia and IIc. In the domain tree, the WRKY domains of the subgroup Ia OnWRKY4 were clustered in the IaN and IaC clades, respectively. In the full protein sequence domain tree, OnWRKY4, was found in the IIc3 clade. Also, in the domain tree, among the 22 group IIc proteins, OnWRKY53.2 and −90 were found in the IaC clade, and OnWRKY57 and −106 in the IaN clade (Fig. 7). In the full protein sequence tree, OnWRKY53.2 and −90 were similarly clustered into the subgroup Ia clade. However, OnWRKY57 and −106 were clustered into IIc3.

### 3.10. Promoter analysis of *OnWRKY* genes

WRKY genes regulate plant responses to various abiotic and biotic stresses that are mediated by several plant hormones.^57^ Analyses of the *OnWRKY* promoters help predict which *OnWRKY* genes could be responsive to these hormones and stresses. The promoter regions of the *OnWRKY* genes were extracted and queried for known DNA *cis*-acting motifs involved in the plant stress responses to ABA, SA, and drought. These included the ABA responsive element (ABRE), and its coupling elements (CE1 or CE3),^78^ drought response element,^79^ SA response element,^80^ and the WRKY binding motif (W-box).^9^ Several elements were found on nearly every promoter region with the SA element being the most abundant (Supplementary Fig. S5).

The promoters of *OnWRKY* genes can be divided into six categories (Supplementary Table S5) based on the number and type of *cis*-acting elements. Considering these *cis*-acting elements in the *OnWRKY* promoters, 68 *OnWRKY* genes could be induced by SA and response to the various stresses. Six *OnWRKY* genes could be regulated by *OnWRKY* genes. Because ABRE needs to couple with a CE, another copy of ABRE, or DRE to confer ABA response,^78^^,^^81^ 11 OnWRKY genes might be responsive to ABA, including 6 category 2 *OnWRKY* genes (*OnWRKY* 1, 21, 22, 43, 49, and 125), 4 category 4 genes (*OnWRKY*79, 119, 53.1, and 58) and the sole category 5 gene (Supplementary Table S5). One gene, *OnWRKY1*, contains all elements above, and could response to ABA, SA, drought, and be regulated by *WRKY* genes.

### 3.11. Discussion

The *WRKY* transcription factor family is one of the largest gene families. They are involved in a wide variety of functions including plant development, stress response, senescence, and seed dormancy and germination. They have been identified in all land plants and even green algae, slime mould and protozoa (for a detailed review, see Rushton et al.^9^). Although many *WRKY* genes have been studied in cultivated rice (*O.* *sativa*), the *WRKY* genes in the wild rice species *O.* *nivara*, the direct progenitor of *O.* *sativa*, have not been studied. *O.* *nivara* shows abundant genetic diversity^38^ and elite drought^39^ and disease resistance^82^ features. Thus, research on *O.* *nivara* may lead to the engineering of more robust cultivars of the modern cultivated *O.* *sativa*.

In this study, a total of 97 *OnWRKY* genes were identified by using an HMM model derived from the *OsWRKY* genes. The *OnWRKY* genes were classified based on the number of WRKY domains and features of the zinc finger motifs of WRKY domain sequences.^8^^,^^32^

Although groups I, II, and III WRKYs have been well characterized in many plant species, group IV WRKYs are rarely reported. WKRY proteins that have an incomplete WRKY domain were classified as group IV, as previously reported by Xie et al.^32^ Group IV was reevaluated in this study by comparing WRKY56 and −58 proteins in *O.* *sativa* ssp. *Japonica* and *O.* *nivara*, along with WRKY58 in *O.* *sativa* ssp. *indica* (OsiWRKY56 was not found). This comparison suggested that the *WRKY56* and *−58* genes in these species did lose a part of the WRKY domain (Supplementary Fig. S6), and was not due to mistakes in genome annotations. This also suggests that *WRKY56* and *−58* genes may have lost their function as WRKY transcription factors.

The diversification of the *WRKY* genes into four groups resulted from a long evolution history. Several hypotheses have been proposed to explain how *WRKY* genes evolved. Hypothesis 1 stated that subgroup Ia was the ancestor of other *WRKY* genes.^2^^,^^32^^,^^42^^,^^48^ Hypothesis 2 proposed that subgroup Ia and IIc have a common ancestor, IIc-like genes, and other WRKY group genes evolved from subgroup Ia.^48^ Hypothesis 3 gave two alternative possibilities for the evolution of *WRKY* genes—the “Group I Hypothesis” suggested that group Ia *WRKY* genes are the most primitive and all other *WRKY* genes are derived from the C-terminal domain of the group Ia *WRKY* genes; the “IIa + b Separate Hypothesis” stated that group IIa + b *WRKY* genes evolved directly from the single WRKY domain of the ancestral algae *WRKY* genes, differing from the group I-derived lineage.^55^

**Figure 9:**
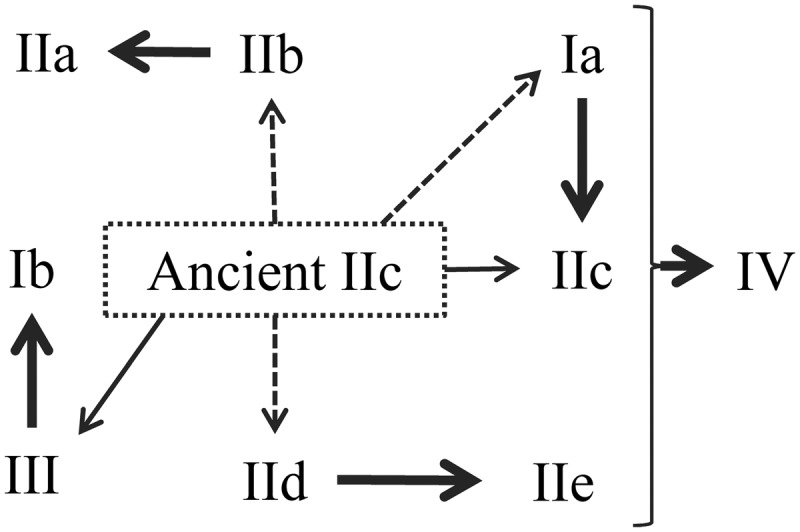
Hypothesis on *OnWRKY* gene evolution. Ancient IIc *WRKY* genes were the ancestors of all *WRKY* genes. The ancient IIc WRKY proteins contain CX_4_C and HXH sites like modern subgroup IIc, but might contain more diversified sequences immediately following the CX_4_C motif. Subgroups Ia, IIb, IIc, IId, and III evolved directly from the ancient IIc *WRKY* genes. Some of the modern subgroup IIc *WRKY* genes evolved directly from the ancient IIc *WRKY* genes, but most IIc *WRKY* genes evolved from the subgroup Ia *WRKY* genes. The subgroup IIa *WRKY* genes evolved from subgroup IIb *WRKY* genes, subgroup IIe from subgroup IId, and subgroup Ib from group III. Group IV genes evolved from various other groups or subgroups by losing part of their WRKY domains. Solid thick arrow: strong evidence; solid thin arrows: some evidence; dash arrow: hypothetical scheme or groups.

All hypotheses could explain the evolution of OnWRKYs, but only partially. Herein, we proposed an overall model of *OsWRKY* gene evolution based on the phylogenetic trees and the sequences with the WRKY domains (Fig. 9). An ancient IIc *WRKY* gene is the ancestor of all *WRKY* genes. Group IIc *WRKY* genes are most diverse. Phylogenetic analyses of OnWRKY domains show that the subgroup IIc domains were clustered into two clades (Fig. 7). However, the bootstrap values for clade IIc1 and IIc2 were extremely low (<20). Furthermore, the topology of the full protein sequence tree is similar to that of the domain tree regarding most clades. But subgroup IIc OnWRKYs were clustered into three separate clades in the full protein sequence tree, indicating they are paraphyletic.

It is generally believed that group IIc WRKYs evolved from group Ia WRKYs.^2^^,^^32^^,^^48^ Some of our data support this hypothesis. First, the C-terminal WRKY domains of group Ia proteins are very similar to the subgroup IIc proteins (Supplementary Fig. S2). They are all the same length and many of the amino acids align identically. Most IIc WRKYs have a PR intron (20/23) and all group Ia C-terminal WRKY domains have a PR intron (11/11). Second, subgroup IIc OnWRKY53.2 and −90 WRKY domains were clustered into the IaC clade in the domain tree and the Ia clade in the full protein tree (Figs. 7 and 8). This suggests that OnWRKY53.2 and −90 may have derived from subgroup Ia by losing N-terminal WRKY domains. However, there is some evidence that contradicts this hypothesis. Most of the subgroup IIc WRKY proteins in clade IIc1 contain a KVE or RVE sequence in their zinc finger motifs. Most group IaC WRKYs contain an HVE sequence in their zinc finger. The exceptions are subgroup IIc OnWRKY53.2 and −90 which contain the HVE sequence (Supplementary Fig. S2). This HVE sequence may be the reason they cluster with group IaC in the phylogenetic tree. The close relationship between subgroup IIc1 and group Ia may indicate that group Ia evolved from group IIc1. It may be the case that OnWRKY53.2 and −90 evolved from modern subgroup Ia WRKYs.

The case regarding OnWRKY57 and −106 is more complicated. Based on the domain tree, these two proteins cluster with clade IaN. However, based on the full protein sequence tree, it appears that they may have evolved from an ancient IIc protein (Fig. 9). This hypothesis is further supported by the observation that among all IIc proteins in the IaN clade (Fig. 7), *OnWRKY57* and *−106* do not contain PR-type introns in the coding regions for the WRKY domains (Supplementary Fig. S2).

Given the data observed, we hypothesize that an ancient subgroup IIc *WRKY* gene was the original progenitor of WRKY genes (Fig. 9). Previous hypotheses state that group III WRKYs evolved from subgroup Ia and IId,^48^^,^^55^ However, we hypothesize that Group III OnWRKYs diverged first from ancient subgroup IIc based on phylogenetic analyses. Analyses of a moss and a green alga also support this hypothesis. Group III WRKYs in the moss *Physcomitrella patens*^83^ and the green alga *Ostreococcus lucimarinus*^84^ share the same CX_4_C pattern in their C_2_H_2_ zinc finger motifs with subgroup IIc WRKYs in *O.* *nivara* and all other *WRKY*-gene containing organisms. If group III WRKYs were derived from subgroup Ia, then subgroup Ia WRKYs would have contained C_2_HC zinc fingers. However, all known group Ia WRKYs contain two C_2_H_2_ zinc fingers and all group III WRKYs contain C_2_HC zing fingers. We also think that it is unlikely that group III *WRKY* genes evolved from group IId because the alga *O.* *lucimarinus* contains only two *WRKY* genes, belonging to subgroup IIc and group III, respectively.^84^^,^^85^ However, the oldest group IId gene was found in the moss, *P.* *patens.*

Based on the phylogenetic analysis, subgroups Ia, IIb, and IId may also have evolved from ancient IIc *WRKY* genes. Group IV *WRKY* genes could be derived from any other groups of *WRKY* genes by losing part of a WRKY domain (Fig. 9). Further research is necessary to verify their evolutionary relationships.

*WRKY* genes expanded and diversified in the evolutional process from green algae to land plants, expanded and diversified more rapidly in seed plants.^42^^,^^55^ Green algae usually contain just a few *WRKY* genes, but angiosperm plants often contain more than 100 WRKY genes. The published WRKY profiles showed that Subgroup IIc *WRKY* genes expanded more rapidly in dicots and group III *WRKY* genes expanded more rapidly in monocots.^7^ The mechanism by which the number of WRKY genes within a species expanded is interesting. Segmental and tandem gene duplication plays an important role in Arabidopsis.^86^ Tandem gene duplication was also reported in *Brachypodium* distachyon.^6^ Similar *WRKY* gene arrangements were found in *O.* *nivara*. In total, 17 *OnWRKY* genes were considered as tandem duplication genes in three clusters on chromosomes 1, 5, and 11 (Supplementary Fig. S1). Further research demonstrated that clusters 1–3 are the same as those in *O.* *sativa* ssp. *japonica*, which has a fourth cluster that was not found in *O.* *nivara* and appears to be derived from cluster 3. Interestingly, most of these tandem duplication *WRKY* genes belong to group III, suggesting group III WRKY genes possibly expanded mainly by gene tandem duplication in rice.

The expanded and diversified *WRKY* genes are involved in responding to various stresses and in various developmental processes in plant.^9^^,^^10^^,^^12^^,^^13^^,^^16^^,^^17^^,^^26^^,^^27^^,^^42^ The analysis of the *cis*-acting elements in the *OnWRKY* promoters show that 68 *OnWRKY* genes might be responsive to SA, with 35 genes of them also to ABA and drought. It was reported that *OsWRKY13*, *−31*, *−45*, *−53*, *−71*, and *−89* responded to various pathogenic fungi.^10^ All promoters of their orthologues in *OnWRKYs* contain SARE implying they have similar functions. RNA-sequencing experiments need to be conducted to address the regulation of the *OnWRKY* genes by plant hormones and their expression profiles in response to biotic and abiotic stresses. These data will help elucidate the functions of *OnWRKY* genes and the evolutionary scheme of their promoters.

In summary, we have identified 97 *OnWRKY* genes in the wild rice *O.* *nivara*. The *OnWRKYs* were classified into four groups based on the number of WRKY motifs and features of the zinc finger motifs. Our results demonstrate that group IV *WRKY* genes are not due to incorrect annotations of the genomes, but rather they reflect the evolution of *WRKY* genes. Expression analysis via RNA-seq shows that though many OnWRKY genes were expressed in all tested samples, expression was the lowest in panicles and highest in the roots. About 90% OnWRKY proteins contain potential MAPK phosphorylation sites, suggesting that the functions of most OnWRKYs are regulated by phosphorylation. According to the phylogenetic analyses, we hypothesize that ancient subgroup IIc *WRKY* genes were the ancestors of all *WRKY* genes. Though most of the modern subgroup IIc *OnWRKY* genes were derived from subgroup Ia, A few subgroup IIc *OnWRKY* genes and group III On*WRKY* genes evolved directly from ancient IIc *WRKY* genes. Tandem gene duplication events may account for the expansion mechanisms of some *WRKY* genes, especially for the group III *WRKY* genes, in monocot plants. Together this study provides a solid foundation to the investigation of the functions and evolution of OnWRKY genes.

## Supplementary data

Supplementary data are available at www.dnaresearch.oxfordjournals.org.

## Funding

This study was supported by the “Senior Visiting Scholar Program Abroad of Shandong University of Technology” to H.X. and grant 2007-35304-18279 from the United States Department of Agriculture to Q.J.S. Funding to pay the Open Access publication charges for this article was provided by University of Nevada Las Vegas, USA.

## Conflict of interest

None declared.

## Supplementary Material

Supplementary Data
