# Supplementary material for: WRKY transcription factor genes in wild rice Oryza nivara
Source: DNA Res. 2016 Jun 26;23(4):311–23. doi: 10.1093/dnares/dsw025 (PMC4991837; doi:10.1093/dnares/dsw025)
Supplement: Supplementary Data [file supp_23_4_311__index.html]

Supplementary Data 

# WRKY transcription factor genes in wild rice *Oryza nivara*

## Supplementary Data

files

- Supplementary Data - zip file
