# Supplementary material for: WRKY transcription factor genes in wild rice Oryza nivara
Source: DNA Res. 2016 Jun 26;23(4):311–23. doi: 10.1093/dnares/dsw025 (PMC4991837; doi:10.1093/dnares/dsw025)
Supplement: Supplementary Data [file supp_dsw025_suppl_data.zip › Supplemental Figures 0504.pdf]

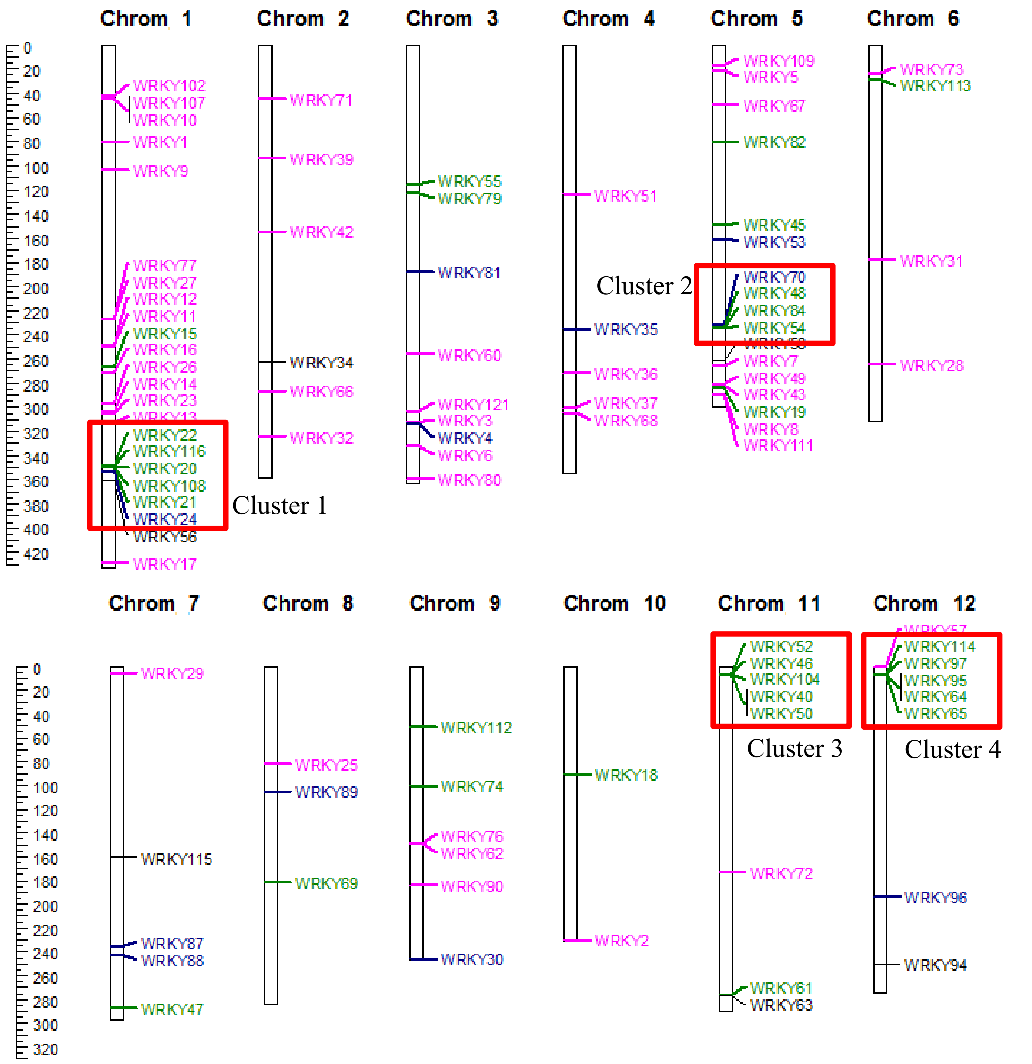

Figure S1 Distribution of *OsWRKY* genes on the *Oryza sativa* chromosomes. The vertical columns represent chromosomes with the gene names shown on the right. The unit on the ruler is 100 kilobase pairs. The chromosome sizes were reduced by a factor of 10^5. The color codes for genes are: blue, group I; pink, group II; green, III; black group IV. WRKY gene clusters are labelled with red boxes.

```
Group Ⅰa                 10        20        30        40        50        60
               ....|....|....|....|....|....|....|....|....|....|....|....|
OnWRKY24C      ILDDGYRWRKYGQKVVKGNPNPR*SYYKCTTAGCPVRKHVERASHDLRAVITTYEGKHNH 60
OnWRKY30C      ILDDGYRWRKYGQKVVKGNPNPR*SYYKCTHPGCSVRKHVERSSHDLKSVITTYEGKHNH 60
OnWRKY35C      ILDDGYRWRKYGQKVVKGNPNPR*SYYKCTHPGCLVRKHVERASHDLKSVITTYEGKHNH 60
OnWRKY4C       LLDDGYRWRKYGQKVVKGNPRPR*SYYKCTADGCNVRKQIERASADPKCVLTTYTGRHNH 60
OnWRKY53C      ILDDGFRWRKYGQKVVKGNPNPR*SYYKCTTVGCPVRKHVERASHDTRAVITTYEGKHNH 60
OnWRKY70C      ILDDGYRWRKYGQKVVKGNPNPR*SYYKCTTAGCPVRKHVERASNDLRAVITTYEGKHNH 60
OnWRKY81C      LLDDGYRWRKYGQKVVKGNPHPR*SYYKCTYQGCDVKKHIERSSQDPKAVITTYEGKHSH 60
OnWRKY87C      ILDDGYRWRKYGQKVVKGNPNPR*SYYKCTNTGCPVRKHVERASHDPKSVITTYEGKHNH 60
OnWRKY88C      LLDDGYRWRKYGQKVVKGNPYPR*SYYKCTYLGCDVKKQVERSVEEPNAVITTYEGKHIH 60
OnWRKY89C      KTSDGYRWRKYGQKIVKGNPNPR*SYYRCTHDGCPVRKHVEKAPDDDNNIVVTYEGKHNH 60
OnWRKY96C      LLDDGYRWRKYGQKVVKGNPHPR*SYYKCTYAGCNVRKHIERASSDPKAVITTYEGKHNH 60
OnWRKY24N      SSDDGYNWRKYGQKQVKGSENPR-SYYKCTFPNCPTKKKVERSL-DGQITEIVYKGTHNH 58
OnWRKY30N      PAEDGYNWRKYGQKQVKNSEHPR-SYYKCTFTNCAVKK-VECSQ-DGQITEIVYKGSHNH 57
OnWRKY35N      VAEDGYSWRKYGQKQVKHSEYPR-SYYKCTHASCAVKKKVERSH-EGHVTEIIYKGTHNH 58
OnWRKY4N       PAKDGYNWRKYGQKQLKDAESPR-SYYKCTRDGCPVKKIVERSS-DGCIKEITYKGRHSH 58
OnWRKY53N      KLEDGYNWRKYGQKQVKGSENPR-SYYKCTYNGCSMKKKVERSLADGRITQIVYKGAHNH 59
OnWRKY70N      SSDDGYNWRKYGQKQMKGSENPR-SYYKCTFPGCPTKKKVEQSP-DGQVTEIVYKGAHSH 58
OnWRKY81N      PADDGYNWRKYGQKAVKGGEYPR-SYYKCTHLSCPVKKKVERSS-DGQITQILYRGQHNH 58
OnWRKY87N      SAEDGYNWRKYGQKHVKGSENPR-SYYKCTHPNCDVKKLLERSL-DGQITEVVYKGRHNH 58
OnWRKY88N      PTDDGYNWRKYGQKAVKGGEYPK-SYYKCTHLNCLVRKNVEHSA-DGRIVQIIYRGQHTH 58
OnWRKY89N      MVGDGFNWRKYGQKQVKSSENSR-SYYRCTNSNCLAKKKVEHCP-DGRVVEIIYRGTHNH 58
OnWRKY96N      PADDGYNWRKYGQKVVKGSDCPR-SYYKCTHPNCPVKKKVEHAE-DGQISEIIYKGKHNH 58
```

```
Group Ⅱc                 10        20        30        40        50        60
               ....|....|....|....|....|....|....|....|....|....|....|....|.
OnWRKY106      TEYDGYNWRMCGQKLVQG-GCHQK-FYYECSQANCGAEKSVTRSA-DGQIKKTVCKGSHNH 58
OnWRKY57       LSYDGYSWRKYGQKQVKG-SEFPR-SYYKCTHPTCPVKRKVEMT-PDGRIAEIVYNGEHNH 58
OnWRKY10       VLDDGFKWRKYGKKAVKS-SPNPR-NYYRCSAAGCGVKKRVERDGDDPRYVVTTYDGVHNH 59
OnWRKY90       ILDDGYRWRKYGQKVVKG-NPNPR*SYYKCTHQGCSVRKHVERASHDLKSVITTYEGKHNH 60
OnWRKY53.2     ILDAGFRWRKYGQKVVKG-NPNPR*SYYKCTTVGCPVRKHVERALHDTRAVITTYAGAVVQ 60
OnWRKY17       LADDGYKWRKYGQKSIKN-SPNPR*SYYRCTNPRCNAKKQVERAVDEPDTLIVTYEGLHLH 60
OnWRKY72       ILDDGYRWRKYGQKAVKN-NKFPR*SYYRCTHQGCNVKKQVQRLSRDETVVVTTYEGTHTH 60
OnWRKY23       ILDDGYRWRKYGQKAVKN-SKHPR*SYYRCTHHTCNVKKQVQRLAKDTSIVVTTYEGVHNH 60
OnWRKY58       ILDDGYRWRKYGQKAVKN-SDFPR*SYYRCTHHTCNVKKQVQRLAKDRGIVVTTYEGVHNH 60
OnWRKY102      VLDDGYKWRKYGQKVVKN-TQHPR*SYYRCTQDNCRVKKRVERLAEDPRMVITTYEGRHVH 60
OnWRKY77       VLDDGYRWRKYGKKMVKN-SPNPR*NYYRCSSEGCRVKKRVERARDDARFVVTTYDGVHNH 60
OnWRKY8        HLEDGYRWRKYGQKAVKN-SSYPR*SYYRCTAPRCGVKKRVERSEQDPSMVITTYEGQHTH 60
OnWRKY11       HLEDGYRWRKYGQKAVKN-SPYPR*SYYRCTTPKCGVKKRVERSYQDPSTVITTYEGQHTH 60
OnWRKY16       HLEDGYRWRKYGQKAVKN-SPYPR*SYYRCTTQKCPVKKRVERSYQDPAVVITTYEGKHTH 60
OnWRKY60       MPEDGYEWKKYGQKFIKNIQKN-R*SYFRCRDQRCGAKKKVEWHPHDPGLLRVVYDGAHHH 60
OnWRKY3        HLEDGYRWRKYGQKAVKN-SPFPR*SYYRCTNSKCTVKKRVERSSDDPSVVITTYEGQHCH 60
OnWRKY34       VLDDGYKWRKYGQKVVKN-SLHPR*SYFRCTHSNCRVKKRVERLSTDCRMVITTYEGRHTH 60
OnWRKY36       VLDDGYKWRKYGQKVVKN-SLHPR*SYYRCTHNNCRVKKRVERLSEDCRMVITTYEGRHTH 60
OnWRKY26       ILDDGYKWRKYGKK----------*NYYRCSTEGCNVKKRVERDKNDPRYVVTTYEGIHNH 51
OnWRKY67       ILDDGFKWRKYGKKAVKN-SPNPR*NYYRCSTEGCNVKKRVERDREDHRYVITTYDGVHNH 60
OnWRKY7        ILDDGYKWRKYGKKSVKN-SPNPR*NYYRCSTEGCNVKKRVERDKDDPSYVVTTYEGTHNH 60
OnWRKY29       HLEDGYRWRKYGQKAVKN-SPFPR*SYYRCTNSKCTVKKRVERSSDDPSVVITTYEGQHSH 60
OnWRKY49       HLEDGYRWRKYGQKAVKN-SPFPR*SYYRCTTQKCPVKKRVERSYQDAAVVITTYEGKHTH 60
```

Figure S2. The alignment of OnWRKY domains. The sequences of OnWRKY domains were classified, and aligned using MUSCLE. About 60 amino acids of WRKY domain were shown. The first arrow marks the beginning of the conserved WRKY motif. The second arrow indicates the conserved motif downstream of the second C in the zinc finger motif. The red stars label the positions where an intron (i.e. the PR intron described in the main text) interrupts the coding sequence. N stands for N-terminus while C represents C-terminus.

```
Group II d+e            10        20        30        40        50        60
               ....|....|....|....|....|....|....|....|....|....|....|....|
OnWRKY6        SDNYSWRKYGQKPIKGSPHPR*GYYRCSSKKDCPARKHVERCRSDPAMLLVTYENEHN-H 59
OnWRKY51       ADDFSWRKYGQKPIKGSPFPR*GYYKCSTLRGCPARKHVERDPADPSMLIVTYEGEHR-H 59
OnWRKY68       PDEYSWRKYGQKPIKGSPYPR*GYYKCSTVRGCPARKHVERATDDPAMLVVTYEGEHR-H 59
OnWRKY42       ADDYSWRKYGQKPIKGSPYPR*GYYKCSTVRGCPARKHVERDPGEPAMLIVTYDGDHR-H 59
OnWRKY94       PDEYSWRKYGQKPIKGSPHPR*GYYKCSSVRGCPARKHVERCVDDPAMLIVTYEGEHN-H 59
OnWRKY121      ADEFSWRKYGQKPIKGSPHPR*GYYKCSSVRGCPARKHVERCVDDPSMLIVTYEGDHN-H 59
OnWRKY109      ADSWSWRKYGQKPIKGSPHLR*GYYKCSSYRGCPARKQVDKCRNDASLLIITYTSDHN-H 59
OnWRKY14       TDSWAWRKYGQKPIKGSPFPR*AYYRCSSSKGCPARKQVERSRNDPDTVIVTYSFEHN-H 59
OnWRKY13       SDSWAWRKYGQKPIKGSPYPR*GYYRCSSSKGCPARKQVERSRADPTVLLVTYSFEHN-H 59
OnWRKY39       ADVWAWRKYGQKPIKGSPYPR*GYYRCSSSKGCPARKQVERSRSDPNTFILTYTGEHN-H 59
OnWRKY66       SDLWAWRKYGQKPIKGSPYPR*GYYRCSSSKGCSARKQVERSRTDPNMLVITYTSEHN-H 59
OnWRKY37       SDLWAWRKYGQKPIKGSPYPR*GYYRCSSSKGCSARKQVERSRTDPNMLVITYTSEHN-H 59
OnWRKY80       SDLWAWRKYGQKPIKGSPYPR*GYYRCSSSKGCMARKQVERSRSDPNMLVITYAAEHN-H 59
OnWRKY12       SDLWAWRKYGQKPIKGSPYPR*GYYKCSSMKGCMARKMVERSPAKPGMLVVTYMAEHC-H 59
OnWRKY111      TDLWAWRKYGQKPIKGSPYPR*GYYKCSSLKACMARKMVERSPEKPGVLVITYIAEHC-H 59
OnWRKY31       PDSWAWRKYGQKPIKGSPYPR-GYYRCSSNKNCAARKQVERCRLDPSFLLLTYTGAHSGH 59
OnWRKY2        SDLWAWRKYGQKPIKGSPYPR-GYYRCSSSKGCSARKQVERSRADPTMLVVTYTSDHN-H 58
OnWRKY25       ADEYSWRKYGQKPIKGSPYPR-GYYRCSTVKGCPARKHVERAADDPATLVVTYEGDHR-H 58

Group II a+b            10        20        30        40        50        60
               ....|....|....|....|....|....|....|....|....|....|....|....|
OnWRKY27       PT---------*KTAKGNPWPRGYYRCTGAPGCPVKKQ-VQRCNHDTSVLVTTYDGVHNH 50
OnWRKY107      MNDGCQWRKYGQKISKGNPCPRAYYRCTVAPNCPVRKQ*VQRCADDMSILITTYEGTHSH 60
OnWRKY73       MNDGCQWRKYGQKIAKGNPCPRAYYRCTVAAGCPVRKQ*VQRCADDMSILITTYEGTHNH 60
OnWRKY1        IADGCQWRKYGQKMAKGNPCPRAYYRCTMATGCPVRKQ*VQRCAEDRSILITTYEGTHNH 60
OnWRKY9        ISDGCQWRKYGQKMAKGNPCPRAYYRCTMAIGCPVRKQ*VQRCAEDKTVLITTYEGNHNH 60
OnWRKY32       MNDGCQWRKYGQKVAKGNPCPRAYYRCTVAPGCPVRKQ*VQRCLEDMSILVTTYEGTHNH 60
OnWRKY5        ISDGCQWRKYGQKMAKGNPCPRAYYRCTMASQCPVRKQ*VQRCAEDKSILITTYEGTHSH 60
OnWRKY43       ISDGCQWRKYGQKMAKGNPCPRAYYRCTMAAGCPVRKQ*VQRCAEDRTVLITTYEGNHNH 60
OnWRKY71       VKDGYQWRKYGQKVTKDNPCPRAYFRCSFAPACPVKKK*VQRSAEDNTILVATYEGEHNH 60
OnWRKY62       VKDGYQWRKYGQKVTRDNPYPRAYFRCAFAPSCPVKKK*LQRCAEDRSMLVATYEGEHNH 60
OnWRKY28       VKDGYQWRKYGQKVTKDNPCPRAYFRCSFAPACPVKKK-VQRSADDNTVLVATYEGEHNH 59
OnWRKY76       VKDGYQWRKYGQKVTRDNPSPRAYFRCAFAPSCPVKKK-VQRSAEDSSLLVATYEGEHNH 59
```

Figure S2 (continued) The legend is the same as the above, except that the red stars represent the positions where an intron (i.e. the VQR intron described in the main text) interrupts the coding sequence.

Group III

```
                   10        20        30        40        50        60        70
OnWRKY48     LWRKYGQKEIKN-SKHPR-----------LYYRCSYKDDHGCTATKQVQQSE---------ED-PSI-YVITYFGDHTC 56
OnWRKY21     IWRKYGQKEING-CKHPR-----------LYYRCAFR-GQGCLATRRVQQSQ--------SQDDPAAAFVIAYYGEHTC 58
OnWRKY54     LWRKYGQKEIKN-SKHPR-----------FYYRCSYKDDHGCTATKQVQQSE------TADDDTASPVYIITYFGEHTC 61
OnWRKY69.1   SWRKYGQKDILG-AKHPR-----------GYYRCTHRNTQGCTATKQVQRTD---------DD-ASL-FDVVYHGEHTC 56
OnWRKY82     QWRKYGQKNINN-SNHQR*----------SYYRCSYKHEQNCKATKTVQQLD---------SAGETIMYTVVYYGQHTC 59
OnWRKY115    LWRKYGQKDILG-ARFAR*----------SYYRCAQ--MLGCTARKQVQQSD---------DD-PSR-LEITYIGLHTC 55
OnWRKY47     QWRKYGQKHIQDSPNNPR*----------SYYRCTHRPDQGCMATKQVQTSE---------SN-SSE-FVISYYGEHTC 58
OnWRKY112    EWRKYGQKSISK-TKHSR*----------SYYRCTNQKGQGCMATKTVQQIE----NGNSSNSVVKL-YNVDYFCKHTC 63
OnWRKY95     QWRKYGQKWISR-AKHSR*----------SYYRCANRKVQGCPATKTVQQMD------SSGNGTSKL-FNVDYYGQHTC 61
OnWRKY104    QWRKYGQKWISR-AKHSR*----------SYYRCANSKVQGCPATKTVQQMD------SSGNGTSKL-FNVDYYGQHTC 61
OnWRKY69.2   TWRKYGQKDILG-SRYPR*----------SYYRCTHKNYYGCEAKKKVQRLD---------DD-PFT-YEVTYCGNHTC 57
OnWRKY45     AWRKYGQKEIQN-SKHPK*----------AYFRCTHKYDQMCTAQRQVQRCD---------DD-PAS-YRVTYIGEHTC 57
OnWRKY19     SWRKYGQKDILG-AKYPR*----------AYFRCTHRHTQGCNATKQVQRAD---------GD-PLL-FDVVYLGDHTC 57
OnWRKY113    SWRKYGQKDILG-AKFPR*----------GYYRCTYRNAQGCPATKQVQRSD---------AD-LAV-FDVTYQGAHTC 57
OnWRKY50     QWRKYGQKQIEG-AMYPR*----------SYYRCTNSTNQGCLAKKTVQRNG--------GGG-AAG-YTVAYISEHTC 58
OnWRKY74     SWRKYGQKEILG-AKHPR*----------GYYRCTHRHSQGCMATKQVQRTD---------ED-AMV-FDVIYHGEHTC 57
OnWRKY15     SWRKYGQKDILG-AKYPR*----------AYFRCTHRHTQGCHASKQVQRAD---------GD-PLL-FDVVYHGDHTC 57
OnWRKY79     VWRKYGQKDIQN-SPYPR*----------SYYRCTHKLDQGCGARRQTQRCE---------AD-PSN-YDITYYGEHTC 57
OnWRKY46     QWRKYGEKKLSN-SNFPR*----------FYYRCTYKNDMKCPATKQVQQKD--------TND-PPL-FSVTYFNHHTC 58
OnWRKY52     QWRKYGEKKIQG-THFTR*----------SYFRCTYRDDRGCQATKQIQQKD--------NND-PPM-FQVTYSNEHTC 58
OnWRKY84     HWRKYGEKKILD-SEFRK*----------LYYRCGYSDERKCQAKKYVQQEN--------NKH-PPE-FRVTLTNEHTC 58
OnWRKY116    LWRKYGQKNIKD-SAFPR*----------LYYRCSYREDRQCLASKLVQQEN--------DDD-PPL-YRVTYTYEHTC 58
OnWRKY18     LWRKYGEKKIKN-SSFPR*RWCKTVAIYRLYYRCSYRDDRNCMATKVVQQEN--------DAD-PPL-YRVTYIHPHTC 68
OnWRKY114    QWRKYGEKKIQG-THFTR*----------SYFRCTYRDDRGCQATKQIQQED--------KND-PPM-FQVTYSNEHTC 58
OnWRKY20     QWRKYGQKNIQD-SNYLR*----------LYFKCTFSRERSCAAKKQVQQRD--------AGE-PPM-FLVTYLNEHTC 58
OnWRKY55     QWRKYGEKKINN-CNFPR*----------YYYRCTYKDNMNCPATKQIQQKD--------YSD-PPL-YSVTYYNEHTC 58
OnWRKY108    QWRKYGQKKINN-TNFPR*----------SYYRCSYHRERRCPAQKHVQQRD--------GDDVPAL-HVVVYTHEHTC 59
OnWRKY22     --RSYDQENAM------K*----------CYYRCTYHQDHGCPASKHVEQHN--------SED-PPL-FRVIYTNEHTC 51
OnWRKY40     QWRKYGQKHIKN-SKHPR*----------SYYRCTYRQEEKCKATKTVQQREDLHHANSYNGDHPIM-YTVVYYGQHTC 67
OnWRKY64C    QWRKYGQKHINN-SKHPR*----------SYYRCAYRQEEKCKATKTVQQREDLQHANSYNGDHPVM-YTVVYYGQHTC 67
OnWRKY125C   SWRKYGLKDILG-AKYPR*----------SYFRCTHRNTQGCVATKQIQRRD---------GD-PLL-FDVVYHGDHTC 57
OnWRKY64N    QWRKYGQKQIEG-AMYPR*----------SYYRCTNSTNQGCLAKKTVQRNG---------GG-GAAGYTVAYISEHTC 58
OnWRKY125N   SWVKYGQKDILG-TMYPR*----------SYFRCIHRQTKGCLATKQVQPTD---------DD-HQI-LDVIYYGEHTC 57
```

Figure S2 (continued) The legend is the same as the above

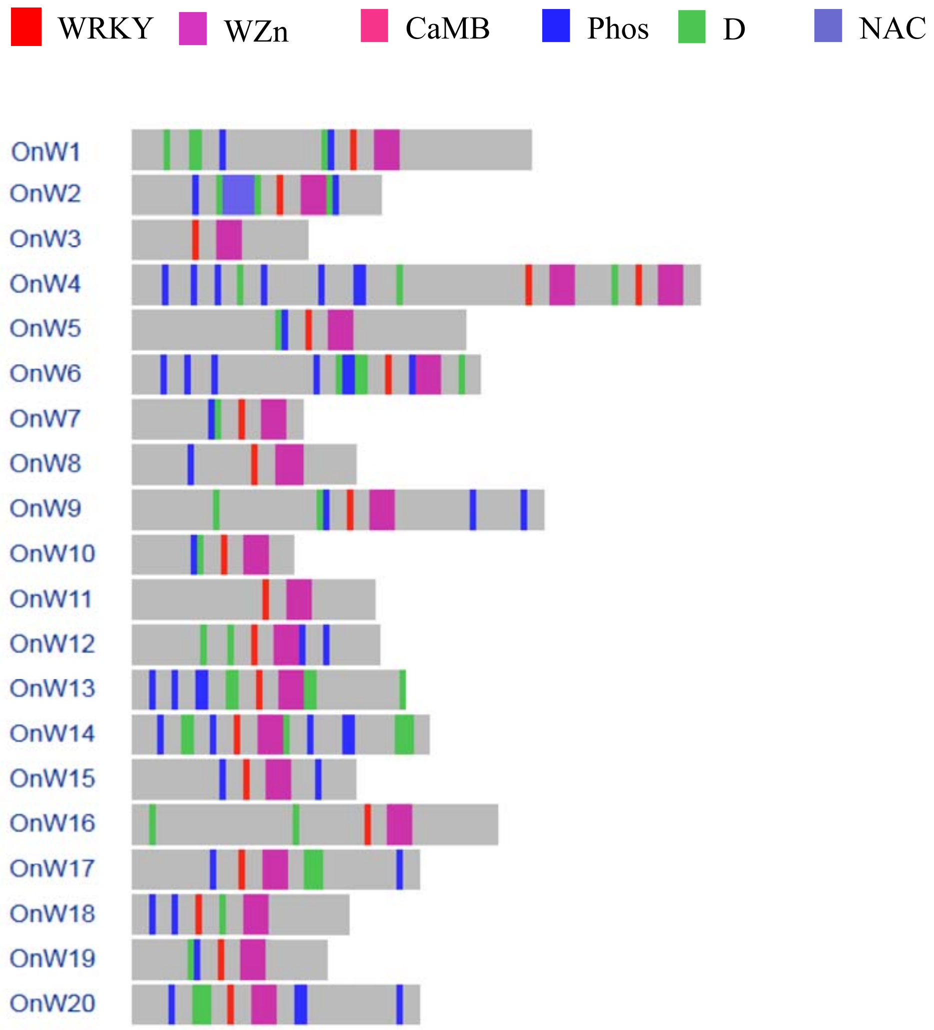

Figure S3. Conserved motifs in OsWRKY peptide sequences. WRKY (red), WRKY motif; WZn (pink), zinc finger motif; CaMB (Violet Red), calmodulin binding motif; Phos (blue), MAPK phosphorylation motif; D (green), docking motif; NAC (Blue Lotus), nascent polypeptide-associated complex.

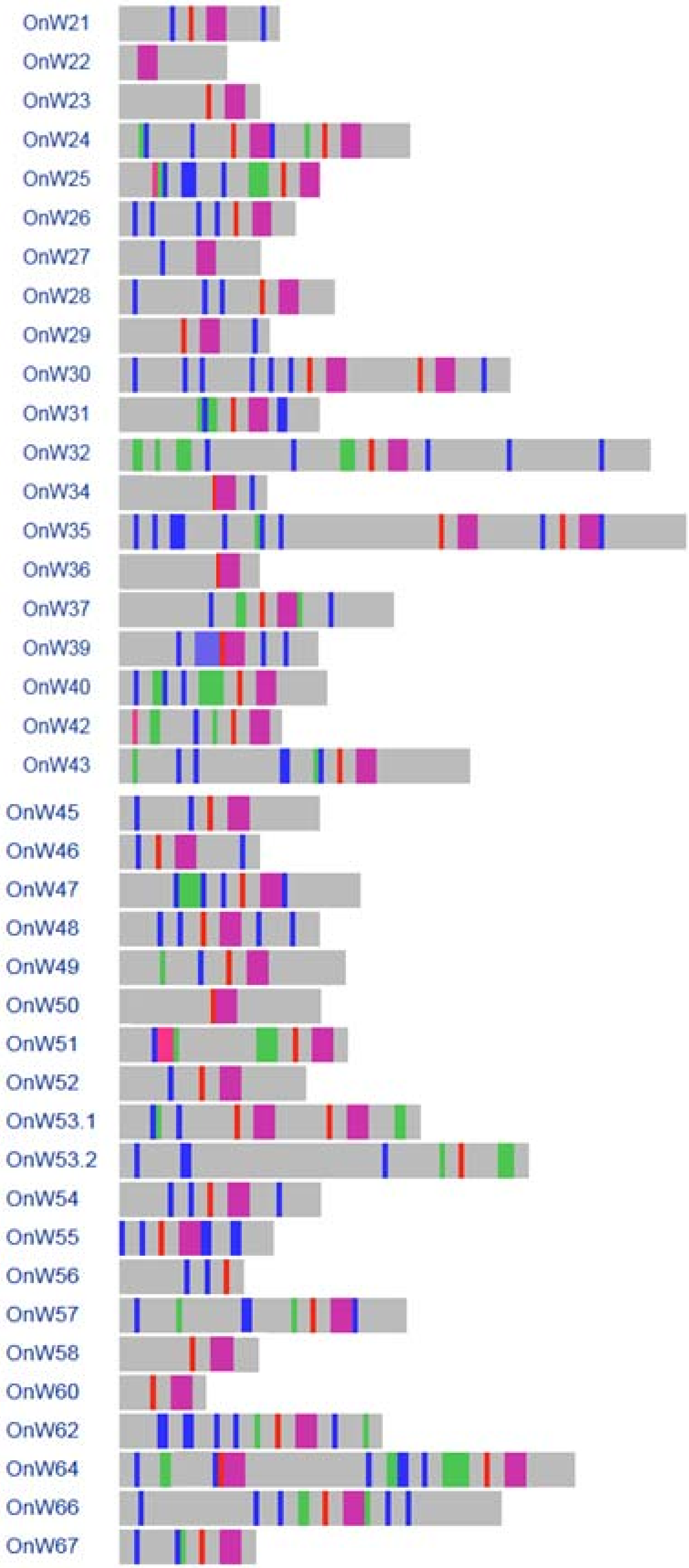

Figure S3 (Continued)

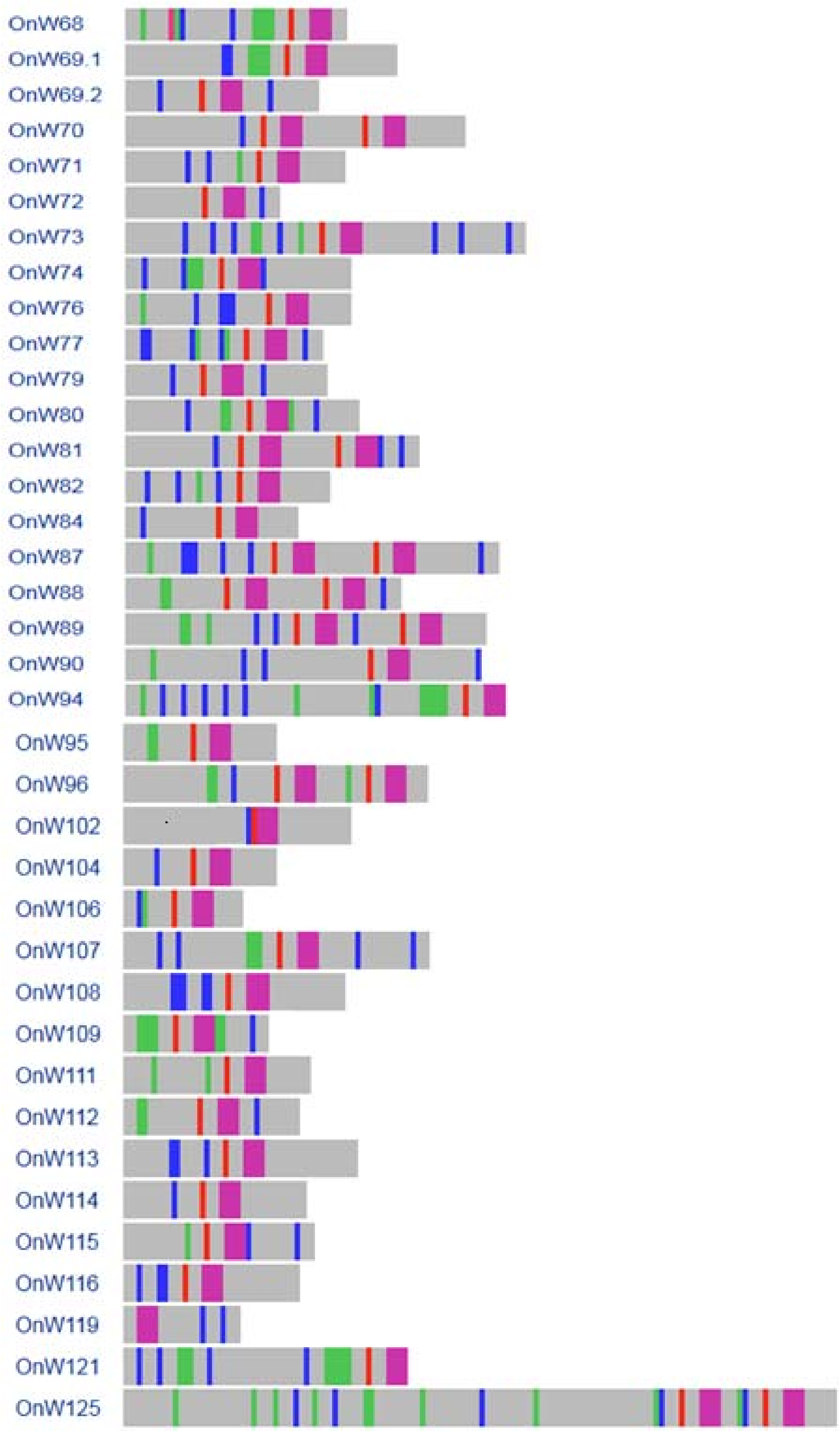

Figure S3 (Continued)

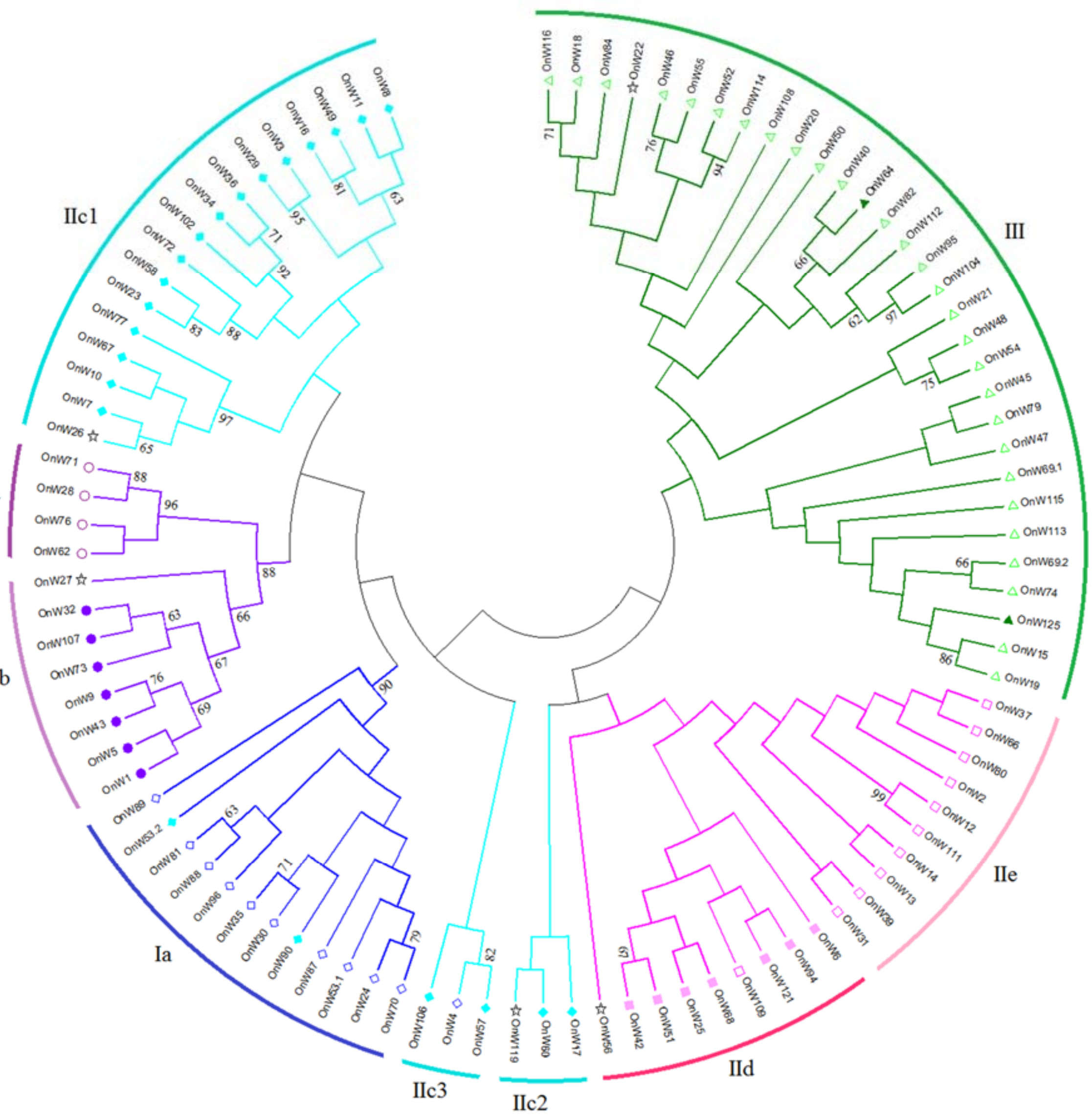

Fig. S4 Phylogenetic analyses of full-length WRKY proteins from *Oryza nivara*. The sequences were aligned by using the MUSCLE method in MEGA6 using the default parameters. The consensus maximum likelihood tree was shown with the results of 100 bootstrap replications. Only bootstrap values ≥ 60 are displayed in nodes. Group Ia, diamond; Group Ib, filled triangle; Group IIa, circle; Group IIb, filled circle; Group IIc, filled diamond; Group IId, filled square; group IIe, square; group III, triangle; Group IV: star.

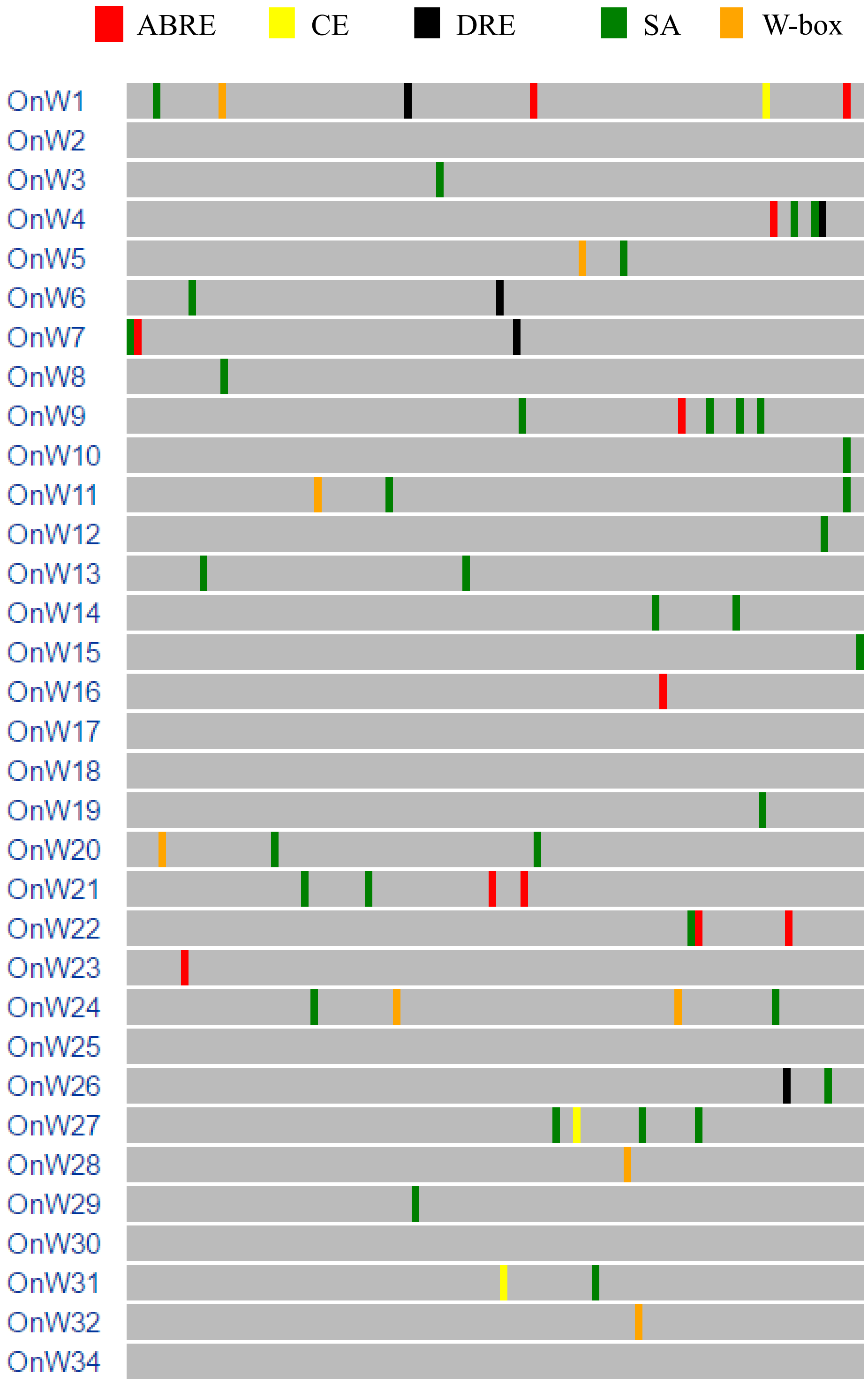

Fig. S5 Fig. S6 Analyses of the 1 kb promoter region (excluding 5' UTR) of *OnWRKY* genes for their potential regulation by ABA, SA and drought. Red bars represent ABRE (ACGTG(G/T)C), yellow bars represent CE1 (TGCCACCGG) or CE3 (GCGTGTC), black bars represent DRE ((T/A/C)ACCGA(C/G)(A/G)), green bars represent SARE (TGACG), and orange bars represent W-box (TTGACC(T/C)).

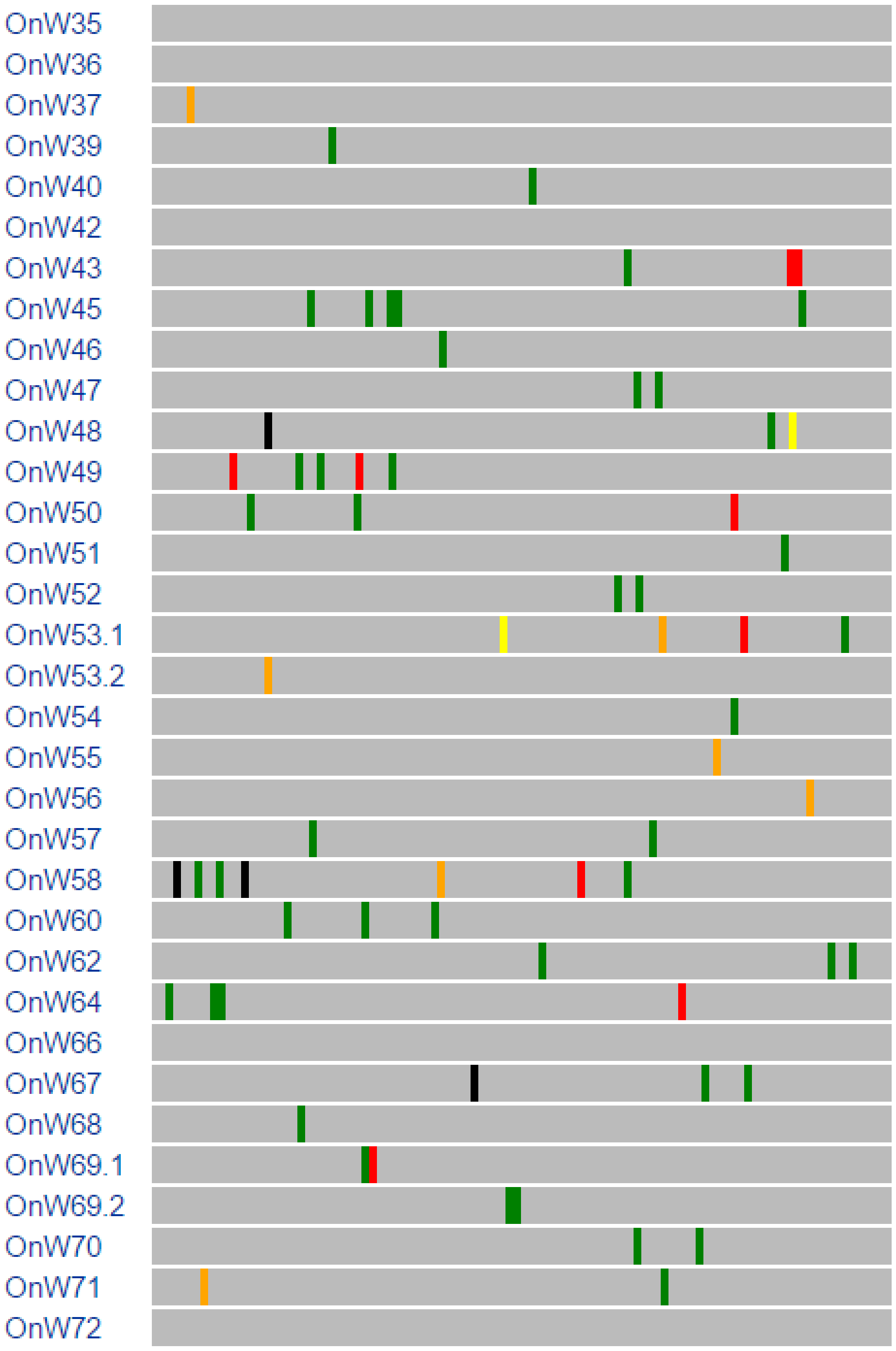

Figure S6 (Continued)

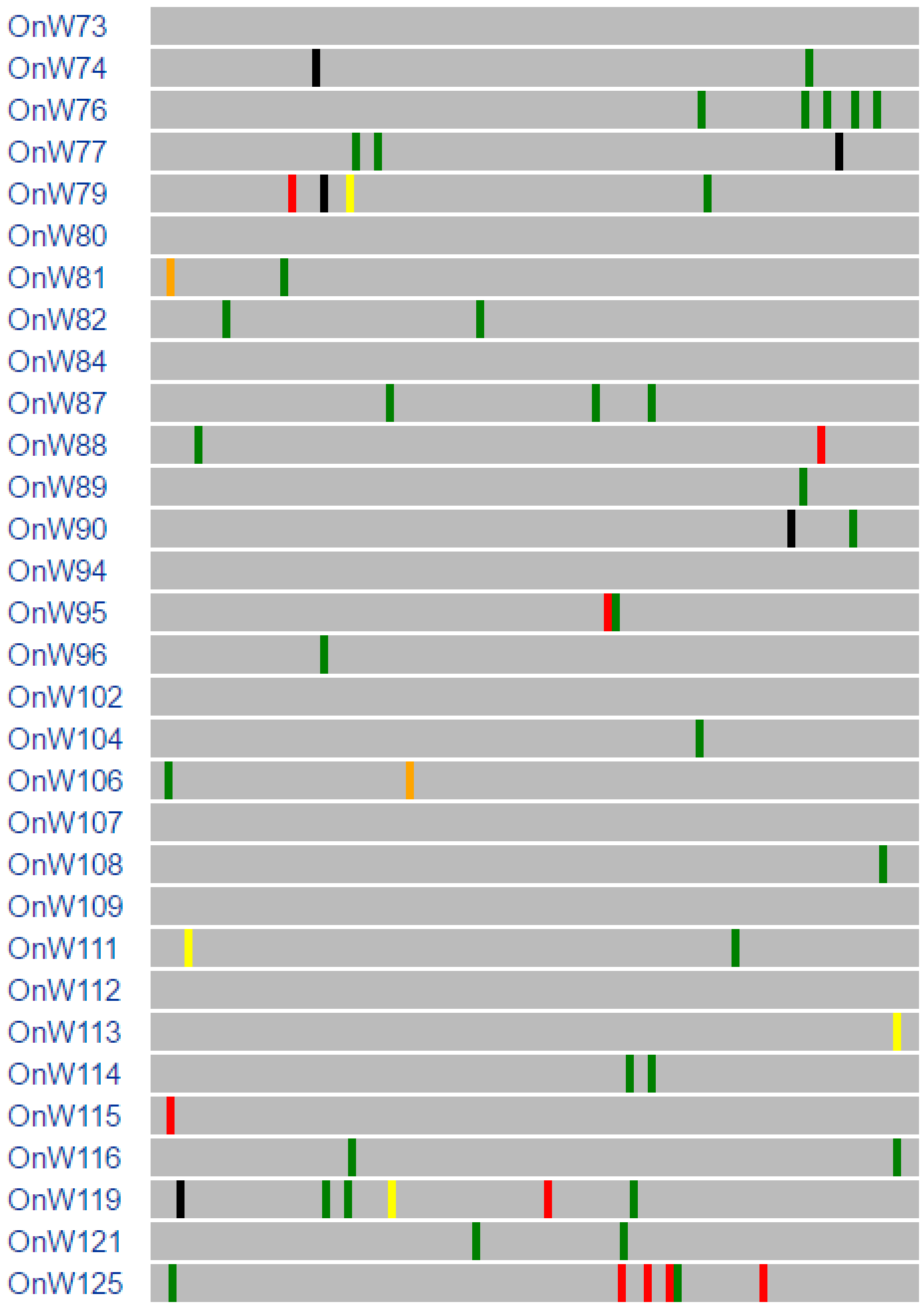

Figure S6 (Continued)

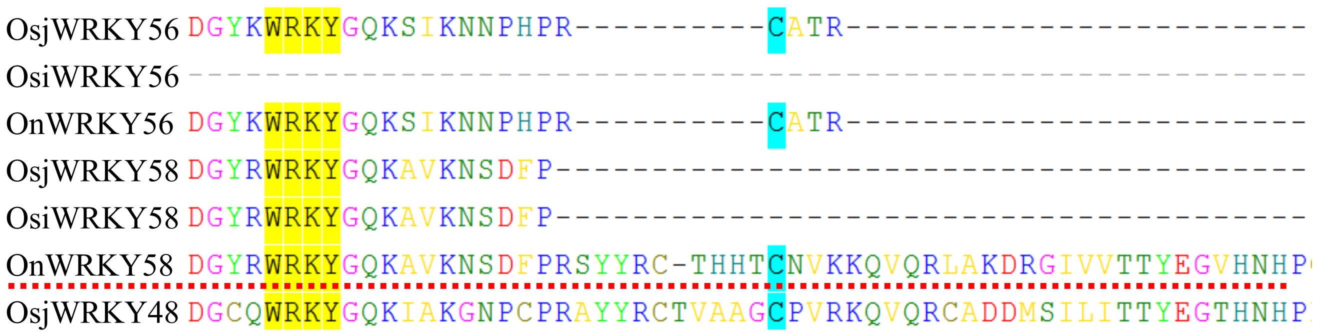

Figure S6. The alignment of partial WRKY domains in WRKY56 and WRKY58 from three species. OsjWRKY48, a subgroup IIb WRKY containing a complete WRKY domain, is included as a reference. Osi: *Oryza sativa ssp. indica*; Osj: *Oryza sativa ssp. japonica;* On: *Oryza nivara.*
